# Cytotoxic hexadepsipeptides and anti-coronaviral 4-hydroxy-2-pyridones from an endophytic *Fusarium* sp.

**DOI:** 10.3389/fchem.2022.1106869

**Published:** 2023-01-12

**Authors:** Shanshan Chang, Biying Yan, Yuchuan Chen, Wuli Zhao, Rongmei Gao, Yuhuan Li, Liyan Yu, Yunying Xie, Shuyi Si, Minghua Chen

**Affiliations:** Institute of Medicinal Biotechnology, Chinese Academy of Medical Sciences & Peking Union Medical College, Beijing, China

**Keywords:** *Fusarium* sp., hexadepsipeptides, 4-hydroxy-2-pyridone, advanced Marfey′s method, anti-coronavirus activity

## Abstract

Three new hexadepsipeptides (**1**–**3**), along with beauvericin (**4**), beauvericin D (**5**), and four 4-hydroxy-2-pyridone derivatives (**6**–**9**) were isolated from the endophytic fungus *Fusarium* sp. CPCC 400857 that derived from the stem of tea plant. Their structures were determined by extensive 1D and 2D NMR, and HRESIMS analyses. The absolute configuration of hexadepsipeptides were elucidated by the advanced Marfey’s method and chiral HPLC analysis. Compounds **4**, and **7**–**9** displayed the cytotoxicity against human pancreatic cancer cell line, AsPC-1 with IC_50_ values ranging from 3.45 to 29.69 μM, and **7** and **8** also showed the antiviral activity against the coronavirus (HCoV-OC43) with IC_50_ values of 13.33 and 6.65 μM, respectively.

## Introduction

*Fusarium*, a common genus of filamentous fungi, is considered as a treasure trove of bioactive secondary metabolites. About 280 compounds, including alkaloids, peptides, amides, terpenoids, quinones, and pyranones have been discovered from the *Fusarium* genus (Li J. et al., 2020; Zhen et al., 2021; Chen et al., 2022; Gu et al., 2022; Zhao et al., 2022). These compounds exhibited a broad spectrum of bioactivities, such as antibacterial, antifungal, antiviral, anti-angiogenic, phytotoxic, and cytotoxic effects (Li M. et al., 2020). Hexadepsipeptides, including typical amide bonds and at least one or two ester bonds, is a typical class of constituents of this genus (Xu et al., 2007; Urbaniak et al., 2020). Beauvericins represent a kind of conventional cyclo-hexadepsipeptides, being composed of three N-methyl amino acids and three hydroxy acid moieties, and displaying a variety of biological activities, such as cytotoxic (Ivanova et al., 2006), antifungal (Fukuda et al., 2004; Zhang et al., 2007), and insecticidal (Urbaniak et al., 2020) activity. Among of them, the beauvericin not only can be used as a co-drug to enhance the antifungal activities of ketoconazole (Nilanonta et al., 2002; Supothina et al., 2004; Zhang et al., 2007), but also exhibited the growth inhibition of human-pathogenic bacteria (Meca et al., 2010). In addition, 4-hydroxy-2-pyridone alkaloids bearing the central 4-hydroxy-2-pyridone moiety linked to two additional substituents at C-3 and C-5 positions are widely distributed in the genus *Fusarium* (Zhan et al., 2007; Jessen et al., 2010). Examples such as (−)-sambutoxin (Kim et al., 1995), (−)-4,6′-anhydrooxysporidinone (Zhan et al., 2007), (−)-oxysporidinone (Jayasinghe et al., 2006), funiculosin (Ando et al., 1969), sambutoxins A and B (Yang et al., 2021), and related annalogues displayed a range of biological activities including antibacterial, antifungal, antiviral, and antitumor properties.

During our ongoing search for the bioactive products from the special habitat fungi (Chen et al., 2019; Li J. et al., 2020; Chang et al., 2022; Wang et al., 2022), *Fusarium* sp. CPCC 400857, an endophytic fungus isolated from a stem of tea plant, was investigated. A chemical investigation on the fungus led to three new hexadepsipeptides (**1**–**3**), along with six known compounds including beauvericin (**4**), beauvericin D (**5**), and four 4-hydroxy-2-pyridinone derivatives (**6**–**9**). The absolute configurations of hexadepsipeptides were assigned by the advanced Marfey’s method and chiral HPLC analysis. Herein, the isolation, structural elucidation, and their cytotoxic and anti-coronavirus activity of the compounds **1**–**9** are described.

## Results

### Structural elucidation of the isolated compounds

Secobeauvericin A (**1**) was obtained as a white amorphous powder. Its molecular formula was assigned as C_45_H_59_N_3_O_10_ by the positive HR-ESI-MS ion [M + H]^+^ at *m/z* 802.4282 (calcd. For C_45_H_60_N_3_O_10_ 802.4279), corresponding for 18 degrees of unsaturation. The NMR spectrum of **1** (Table 1) displayed resonances of three monosubstituted benzenes, three pairs of vicinal methyls, three *N*-methyl groups, three methylenes, nine methines involving six peptidic *α*-methines, together with six amide and/or ester carbonyl carbons (*δ*_C_ 168.7, 168.9, 169.5, 169.7, 171.6, 173.8). A comparison of the molecular composition and NMR data of compounds **1** and beauvericin (**4**) revealed that compound **1** could be a linear hexadepsipeptide (Yuan et al., 2021).

**TABLE 1:** ^1^H NMR (600 MHz) and^13^C NMR (150 MHz) Data for Compounds **1**-**3** in DMSO-*d*_6_ (*δ* in ppm, *J* in Hz).

	No.	1		2		3	
		*δ*_C_, type	*δ*_H_ (*J* in Hz)	*δ*_C_, type	*δ*_H_ (*J* in Hz)	*δ*_C_, type	*δ*_H_ (*J* in Hz)
*N*-Me-Phe	1	169.5, C		169.3, C		169.3, C	
	2	56.8, CH	5.47, m	56.5, CH	5.43, dd (5.4, 12.0)	60.9, CH	4.45, dd (5.4, 11.4)
	3	33.9, CH_2_	3.20, m	34.4, CH_2_	3.03, m; 3.19, m	34.0, CH_2_	3.13, m; 3.01, m
	4	137.0, C		136.7, C		137.6, C	
	5	126.3–129.1, CH	7.13–7.28, m	126.6–129.7, CH	7.13–7.26, m	129.5, CH	7.04–7.28, m
	6	126.3–129.1, CH		126.6–129.7, CH		128.3, CH	
	7	126.3–129.1, CH		126.6–129.7, CH		126.6, CH	
	8	126.3–129.1, CH		126.6–129.7, CH		128.3, CH	
	9	126.3–129.1, CH		126.6–129.7, CH		129.5, CH	
	10-NCH_3_	31.1, CH_3_	2.85, s	31.5, CH_3_	2.94, s	34.9, CH_3_	2.62, s
Hiv	10	173.8, C		169.3, C		168.1, C	
	11	71.7, CH	3.94, d (4.2)	74.8, CH	4.88, d (8.4)	73.7, CH	5.11, d (9.6)
	12	30.5, CH	1.41, m	29.3, CH	1.73, m	29.2, CH	1.91, m
	13	15.5, CH_3_	0.29, d (6.6)	16.8, CH_3_	0.29, d (6.0)	17.8, CH_3_	0.71, d (6.6)
	14	19.1, CH_3_	0.61, d (6.6)	16.3, CH_3_	0.62, d (7.2)	16.9, CH_3_	0.25, d (7.2)
*N*-Me-Phe/Phe	15	169.7, C		170.8, C		169.6, C	
	16	57.0, CH	5.47, m	52.7, CH	4.57, m	55.1, CH	5.50, dd (5.4, 12.0)
	17	33.9, CH_2_	3.26, m	36.6, CH_2_	3.01, m; 2.86, m	34.4, CH_2_	3.05, m
	18	136.9, C		136.8, C		136.4, C	
	19	126.3–129.1, CH	7.13–7.28, m	126.6–129.7, CH	7.13–7.26, m	128.8, CH	7.04–7.28, m
	20	126.3–129.1, CH		126.6–129.7, CH		128.2, CH	
	21	126.3–129.1, CH		126.6–129.7, CH		126.4, CH	
	22	126.3–129.1, CH		126.6–129.7, CH		128.2, CH	
	23	126.3–129.1, CH		126.6–129.7, CH		128.8, CH	
	24-NCH_3_	31.5, CH_3_	2.97, s			31.0, CH_3_	3.08, s
	24-NH				8.32, d (8.4)		
Hiv/HL	24	168.9, C		167.6, C		168.9, C	
	25	74.8, CH	5.07, d (8.4)	78.3, CH	4.66, d (7.8)	67.4, CH	5.20, q (6.6)
	26	28.6, CH	1.49, m	28.9, CH	1.89, m	15.4, CH_3_	1.16, d (6.6)
	27	18.8, CH_3_	0.66, d (7.2)	17.8, CH_3_	0.74, d (6.6)		
	28	15.8, CH_3_	0.30, d (7.2)	18.0, CH_3_	0.62, d (7.2)		
*N*-Me-Phe/Phe	29	171.6, C		170.5, C		169.4, C	
	30	58.9, CH	5.02, m	52.8, CH	4.61, m	55.5, CH	5.58, dd (5.4,11.4)
	31	33.5, CH_2_	3.03, m; 2.93, m	37.0, CH_2_	3.01, m; 2.86, m	33.0, CH_2_	3.12, m; 3.01, m
	32	137.7, C		136.8, C		136.9, C	
	33	126.3–129.1, CH	7.13–7.28, m	126.6–129.7, CH	7.13–7.26, m	128.9, CH	7.04–7.28, m
	34	126.3–129.1, CH		126.6–129.7, CH		128.2, CH	
	35	126.3–129.1, CH		126.6–129.7, CH		126.5, CH	
	36	126.3–129.1, CH		126.6–129.7, CH		128.2, CH	
	37	126.3–129.1, CH		126.6–129.7, CH		128.9, CH	
	38-NCH_3_	32.7, CH_3_	2.87, s			30.9, CH_3_	3.00, s
	38-NH				8.29, d (8.4)		
Hiv	38	168.7, C		168.0, C		168.8, C	
	39	75.4, CH	5.06, d (8.4)	78.4, CH	4.65, d (7.8)	73.2, CH	5.07, d (9.0)
	39-OH		4.15, d (8.4)				
	40	28.7, CH	1.68, m	29.4, CH	1.80, m	29.4, CH	1.74, m
	41	18.6, CH_3_	0.77, d (6.6)	17.7, CH_3_	0.47, d (6.6)	17.9, CH_3_	0.66, d (6.6)
	42	15.9, CH_3_	0.50, d (6.6)	17.7, CH_3_	0.60, d (7.2)	16.3, CH_3_	0.15, d (7.2)

**FIGURE 1:**
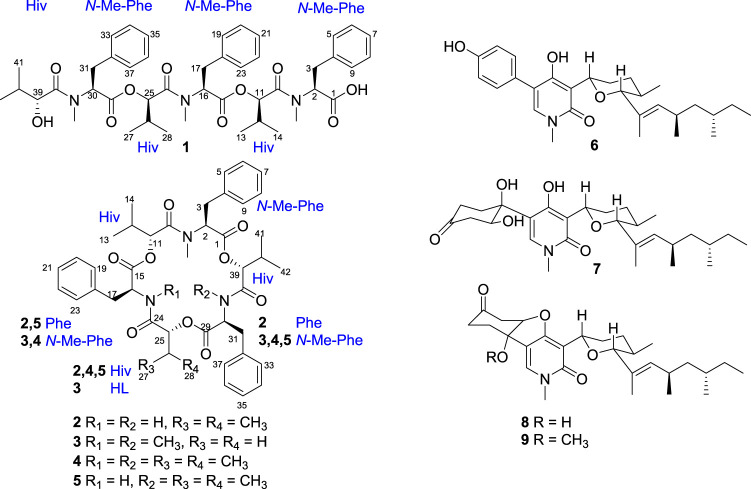
Structures of compounds **1**-**9**.

**FIGURE 2:**
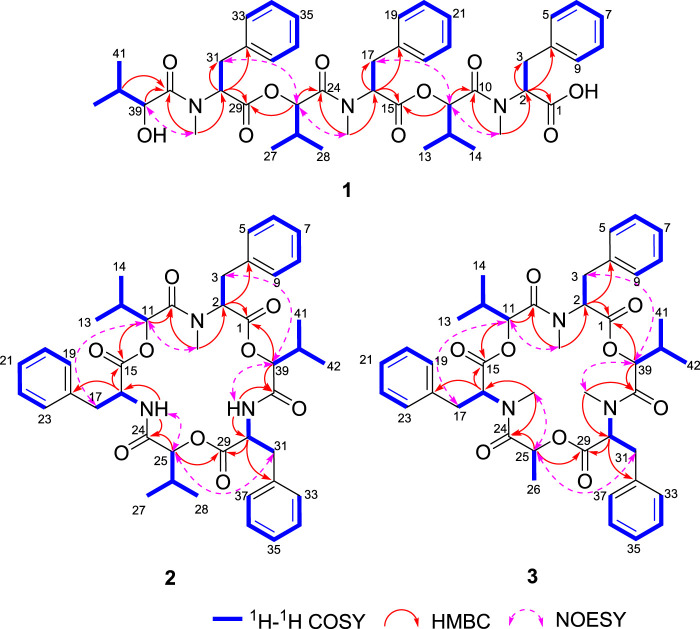
^1^H–^1^H COSY, HMBC, and NOESY correlations of compounds **1**–**3**.

Comprehensive analysis of 2D NMR spectra (Figure 1) revealed the presence of three hydroxyisovaleric acids (Hiv) and three *N*-methyl phenylalanine (*N*-Me-Phe) moieties. HMBC correlations from *N*-CH_3_-10 to C-10, H-11 to C-15, *N*-CH_3_-24 to C-24, H-25 to C-29, and *N*-CH_3_-38 to C-38 established the sequence of Hiv-(*N*-Me-Phe)-Hiv-(*N*-Me-Phe)-Hiv-(*N*-Me-Phe), which was supported by NOESY correlations from *N*-CH_3_-10 to H-11, H-11 to H-17, *N*-CH_3_-24 to H-25, H-25 to H-31, and *N*-CH_3_-38 to H-39. Furthermore, the connection of these hydroxy acid and amino acid residues was confirmed by the HR-MS/MS fragments at *m/z* 663.3252 [M–(*N*-Me-Phe)+OH + Na]^+^, 563.2729 [M–(*N*-Me-Phe)–Hiv + OH + Na]^+^, 402.1890 [M–(*N*-Me-Phe)–Hiv-(*N*-Me-Phe)+OH + Na]^+^, 302.1373 [M–(*N*-Me-Phe)–Hiv–(*N*-Me-Phe)–Hiv + OH + Na]^+^ and 141.0530 [M–(*N*-Me-Phe)–Hiv–(*N*-Me-Phe)–Hiv–(*N*-Me-Phe)+OH + Na]^+^ (Figure 2). Thus, the planar structure of **1** was determined as shown in Figure 1.

**FIGURE 3:**
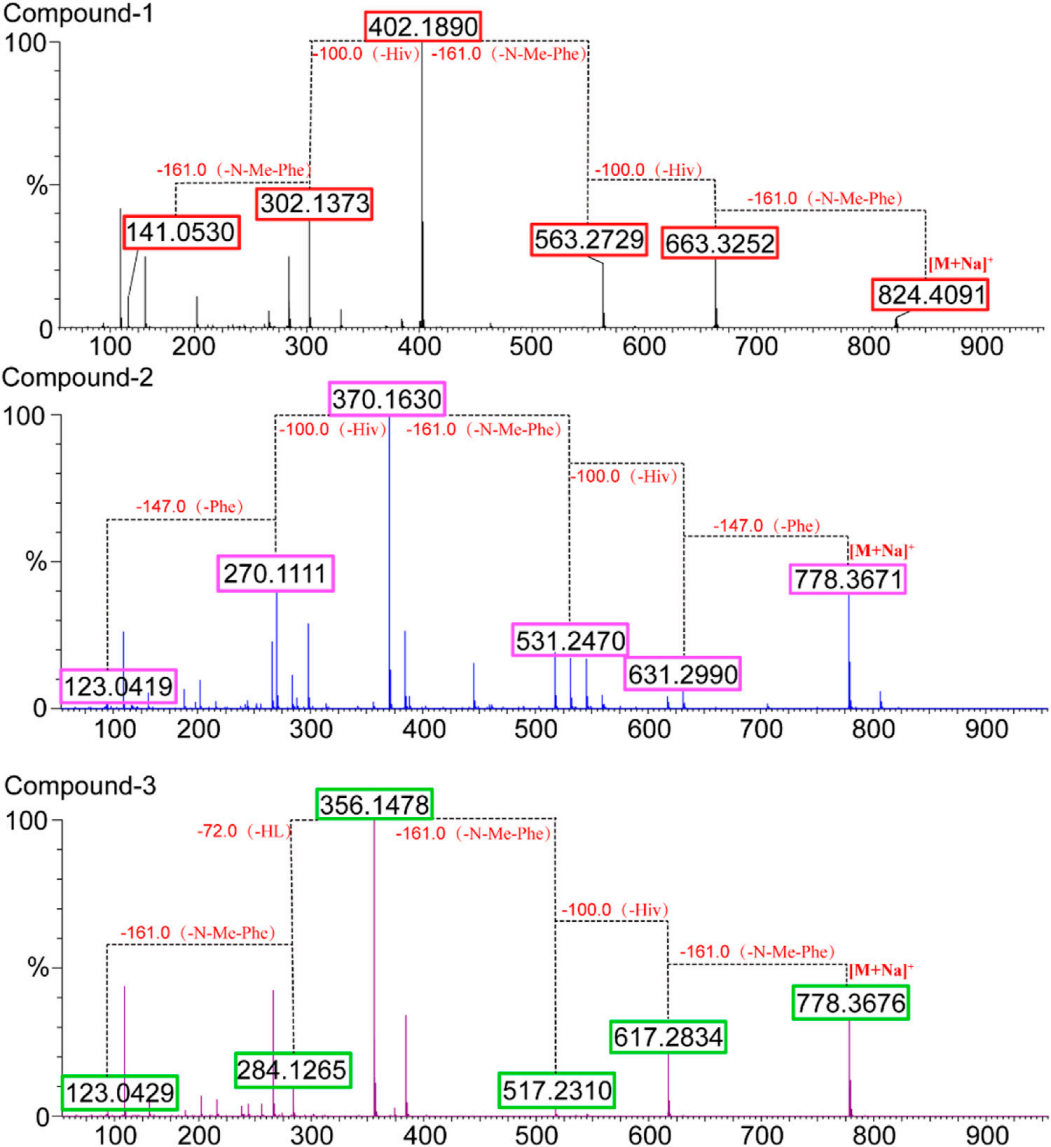
Fragments observed for compounds **1**–**3** b y HR-ESI-MS/MS.

After acid hydrolysis, the absolute configuration of Hiv in **1** was determined to be *R* (Supplemnentary Figure S4; Supplementary Table S2) by chiral HPLC analysis in comparison to the authentic *R*/*S* Hiv units, while the advanced Marfey’s analysis of the hydrolysate of **1** revealed the *N*-Me-Phe residues was *L*-configuration (Supplementary Figure S1) (Tripathi et al., 2009; Wang et al., 2017). Therefore, the structure of compound **1** was determined and designated as secobeauvericin A (Figure 3).

Beauvericin M (**2**) was determined as C_43_H_53_N_3_O_9_, based on the HRESIMS peak at 756.3886 [M + H]^+^ (calcd for C_43_H_54_N_3_O_9_, 756.3860), implying 19 degrees of unsaturation. The NMR data of **2** was closely correlated with beauvericin (4). The extensively analysis of the NMR data (Table 1) revealed that two *N*-CH_3_ in beauvericin were replaced to NH in **2**, which were verified by ^1^H–^1^H COSY correlations of NH-24/H-16/H-17, NH-38/H-30/H-31, together with the HMBC correlations from NH-24 to C-16 and C-24, NH-38 to C-30 and C-38. The above related information shows that compound **2** contained two phenylalanine residues. In addition, the HMBC correlations from *N*-CH_3_-10 to C-10, H-11 to C-15, NH-24 to C-24, H-25 to C-29, NH-38 to C-38, and H-39 to C-1 indicated that the cyclic structure of 2 was Hiv-Phe-Hiv-Phe-Hiv-(*N*-Me-Phe). This connection was confirmed by cross peaks in the NOESY spectra with the correlations *N*-CH_3_-10 to H-11, NH-24 to H-25 and NH-38 to H-39. The cleavage method of the MS/MS spectrometer further verified the above connection of groups (Figure 2). The absolute configuration of *N*-Me-Phe, Phe, and Hiv units were determined as *L*, *L*, and *R* by advanced Marfey’s method and chiral HPLC (Supplementary Figures S2, S4).

Beauvericin N (**3**) was obtained as the white amorphous powder. On the basis of (+)-HRESIMS data, the formula of **3** was established as C_43_H_53_N_3_O_9_. The comparison of NMR data of **3** and **4** suggested that an Hiv unit in **4** was replaced by a 2-hydroxypropionic acid (HL) moiety in **3**, which was verified by the ^1^H–^1^H COSY correlations of H-25/H-26, together with the HMBC correlations from H-26 to C-24. The connection of those *α*-amino acids and *α*-hydroxy acids in **3** was deduced by the HMBC correlations from *N*-CH_3_-10 to C-10, H-11 to C-15, *N*-CH_3_-24 to C-24, H-25 to C-29, *N*-CH_3_-38 to C-38 and H-39 to C-1, as well as NOESY correlations and HRESIMS/MS analysis (Figures 1, 2). The absolute configurations of the *α*-hydroxy acids were determined as *R*-Hiv, *R*-HL by using the chiral HPLC, and the amino acids were assigned as *L*-*N*-Me-Phe by the advanced Marfey’s method. Accordingly, compound **3** was corroborated and named as beauvericin N (Figure 3).

**FIGURE 4:**
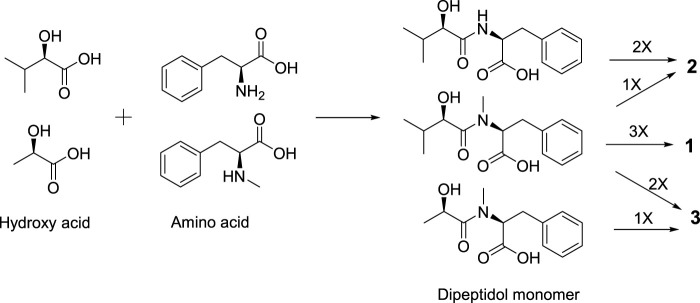
Postulated biogenetic pathway of **1**–**3**.

Secobeauvericin A (**1**) is assembled from three *R*-Hiv-*N*-methyl-*L*-Phe acid dipeptidol monomers. Beauvericin M (**2**) is formed as cyclic combined with two *R*-Hiv-*L*-Phe acid dipeptidol monomers and one *R*-Hiv-*N*-methyl-*L*-Phe acid monomer, while beauvericin N (**3**) is formed as cyclic with two *R*-Hiv-*N*-methyl-*L*-Phe acid dipeptidol monomers and one *R*-HL-*N*-methyl-*L*-Phe acid monomer (Urbaniak et al., 2020) (Figure 4).

The known compounds **4**–**9** were identified as beauvericin (Isaka et al., 2011), beauvericin D (Fukuda et al., 2004), (–)-sambutoxin (Kim et al., 1995), (–)-oxysporidinone (Jayasinghe et al., 2006), fusapyridon A (Wijeratne et al., 2011), and (–)-fusoxypyridone (Jayasinghe et al., 2006) by comparison of MS and 1D NMR data in the literature, respectively.

### Physicochemical properties and spectroscopic data of compounds 1-3

Secobeauvericin A (**1**): White amorphous powder; mp. 90°C–91°C; [α] –73.3 (*c* .4, MeOH); UV (LC): 210 nm; IR *ν*_max_: 3441, 2966, 2933, 2876, 1739, 1659, 1633, 1456, 1285, 1091, 1023, 828, 748, 700 cm^−1^; ^1^H (600 MHz) and ^13^C NMR (150 MHz), see Table 1; HRESIMS *m/z* [M + H]^+^ 802.4282 (calcd for C_45_H_59_N_3_O_10_, 802.4279).

Beauvericin M (**2**): White amorphous powder; mp. 85°C–86°C; [α] +6.7 (*c* .2, MeOH); UV (LC) 217 nm; IR *ν*_max_: 3358, 3279, 2927, 1742, 1679, 1457, 1421, 1203, 1134, 1026, 802, 722, 700 cm^−1^; ^1^H (600 MHz) and ^13^C NMR (150 MHz), see Table 1; HRESIMS *m/z* [M + H]^+^ 756.3886 (calcd for C_43_H_53_N_3_O_9_, 756.3860).

Beauvericin N (**3**): White amorphous powder; mp. 82°C–83°C; [α] +13.3 (*c* .2, MeOH); UV (LC) 217 nm; IR *ν*_max_: 3400, 3306, 2967, 2935, 1738, 1664, 1640, 1457, 1422, 1203, 1134, 1083, 1029, 835, 722, 700 cm^−1^; ^1^H (600 MHz) and ^13^C NMR (150 MHz), see Table 1; HRESIMS *m/z* [M + H]^+^ 756.3830 (calcd for C_43_H_54_N_3_O_9_, 756.3860).

### Cytotoxicity and anti-coronavirus activity compounds 1–9

Beauvericins and 4-hydroxy-2-pyridones were shown to display potent cytotoxic activity against different human cell lines. Therefore, compounds **1**-**9** were evaluated the cytotoxic activity *in vitro*. Compounds **4**, and **7**–**9** showed the cytotoxicity against human pancreatic adenocarcinoma cell line AsPC-1 with IC_50_ values of 3.45, 17.62, 29.69, and 18.81 *μ*M, respectively, while compounds **1**–**3**, **5**, and **6** were inactive at 90 *μ*M (Table 2). Compared with the positive drug, compounds **4**, and **7**–**9** exhibited weaker cytotoxicity than the gemcitabine. In addition, funiculosin could inhibited both the RNA and DNA virus in previous report (Ando et al., 1969). Therefore, the 4-hydroxy-2-pyridones (6–9) were tested for the inhibition against the coronavirus (HCoV-OC43). Compounds **7** and **8** displayed the antiviral activity against the coronavirus (HCoV-OC43) with IC_50_ values of 13.33 and 6.67 *μ*M, and SI values of 1.7 and 1.7, respectively, and showed slightly better than the positive drug ribavirin (Table 3).

**TABLE 2:** Cytotoxicity of compounds 1–9

	IC_50_, *μ*M									Positive control
	1	2	3	4	5	6	7	8	9	Gemcitabine
AsPC-1	>90	>90	>90	3.45	>90	>90	17.62	29.69	18.81	1.53

**TABLE 3:** Anti-coronavirus (HCoV-OC43) activity of compounds 7–9.

Compd	TC_50_ (μg/mL)	IC_50_ (μg/mL)	SI (TC_50_/IC_50_)
6	<0.02	-	-
7	23.09	13.33	1.7
8	11.55	6.67	1.7
9	11.55	>6.67	-
Ribavirin	100	19.24	5.2

## Materials and methods

### Fungal materials

The fungus *Fusarium* sp. CPCC400857 was isolated from a stem of tea plant collected from Shaanxi Province, China. The strain was deposited in the China Pharmaceutical Culture Collection (Institute of Medicinal Biotechnology, Chinese Academy of Medical Sciences and Peking Union Medical College, No. CPCC 400857).

### Fermentation and extraction

The fungal strain was cultured on slants of potato dextrose agar (PDA) at 25°C for 7 days. Subsequently, the spores were used to inoculate in 500 ml Erlenmeyer flasks, each containing 100 ml of potato dextrose broth at 25°C (180 rpm) for 4 days to obtain the seed culture. The large-scale fermentation proceeded in 30 Erlenmeyer flasks (500 mL) containing 100 g of rice and 100 ml of distilled water, which were autoclaved at 121°C for 15 min. After being cooled to room temperature, each flask was inoculated with 5 ml of seed culture and incubated at 25°C for 30 days.

The fermented material was extracted with 95% EtOH (12 L for 2 times) and with 50% EtOH (12 L for 1 time). The solution was combined and evaporated under the reduced pressure to yield an aqueous suspension (4.0 L). The aqueous suspension was partitioned with EtOAc (3 × 4.0 L). The organic solution was concentrated to dryness, and yielded a dark brown extract (124.3 g).

### Isolation and purification

The EtOAc extract (124.3 g) was subjected to silica gel column chromatography using CH_2_Cl_2_/MeOH gradient elution (50:1, 35:1, 25:1, 20:1, 15:1, 10:1, 1:1, and 1:0) to afford 10 fractions (Fr.1−Fr.10).

Fr. 2 (890 mg) was fractionated by Sephadex LH-20 column chromatography with CH_2_Cl_2_/MeOH (1:1) to afford three subfractions (Fr. 2-1–Fr. 2–3). Fr. 2–2 (41.0 mg) was further purified by reversed-phase semipreparative HPLC (Capcell Pak PFP column, 5 *μ*m, 10 × 250 mm, 1.5 mL/min, 60% CH_3_CN/H_2_O) to yield **2** (3.1 mg).

Fr. 5 (1.7 g) was separated to seven subfractions (Fr. 5-1–Fr. 5–7) with reversed-phase (RP) flash column chromatography (5 mL/min, 5%–100% MeOH/H_2_O), then Fr. 5–3 (27 mg) was further purified by reversed-phase semipreparative HPLC (Capcell Pak PFP column, 5 *μ*m, 10 × 250 mm, 1.5 mL/min, 66% CH_3_CN in 0.1% trifluoroacetic acid) to yield **1** (4.1 mg). Subsequently, the purification of Fr. 5–7 (192 mg) with Sephadex LH-20 column chromatography (CH_2_Cl_2_/MeOH, 1:1) yielded to seven subfrations (Fr. 5-7-1–Fr. 5-7-7). Then, the compound **4** (46.6 mg) was purified by reversed-phase semipreparative HPLC (Capcell Pak PFP column, 5 *μ*m, 10 × 250 mm, 1.5 mL/min, 80% CH_3_CN/H_2_O containing 0.1% TFA) from the subfraction Fr. 5-7-1 (72 mg). The subfraction of Fr. 5-7-5 (51 mg) was further isolated by reversed-phase semipreparative HPLC (Capcell Pak MGⅡ column, 5 *μ*m, 10 × 250 mm, 1.5 mL/min, 78% CH_3_CN/H_2_O) to yield **3** (2.7 mg), **5** (2.5 mg), and **9** (3.7 mg).

Fr. 10 (1.3 g) was fractionated by reversed-phase (RP) flash column chromatography with 5–100% MeOH to obtain **8** (3.1 mg) and six subfractions. Then Fr. 10–3 (33 mg) was isolated by reversed-phase semipreparative HPLC (Capcell Pak MGⅡ column, 5 *μ*m, 10 × 250 mm, 1.5 mL/min, 69% CH_3_CN in 0.1% trifluoroacetic acid) to get **6** (9.6 mg), and **7** (4.2 mg).

### Advanced Marfey’s method (Tripathi et al., 2009; Wang et al., 2017)

Each of the compounds **1**–**3** (2 mg) in 1 mL of 6 M HCl were heated at 110°C for 18 h. The crude hydrolysate was divided into three portions and evaporated to dryness separately. Two of them were added to 50 *μ*L of 1% (w/v) *L* and *L*/*D*-FDLA (Marfey’s reagent) and 100 *μ*L 1 M NaHCO_3_ solution, respectively, and the mixtures were incubated at 40°C for 1 h. After being cooled to the room temperature, the reactions were quenched by additional 100 *μ*L 1 M HCl, and diluted with 250 *μ*L MeOH. The *L*- and *L*/*D*-FDLA derivatives were analyzed by LC/MS on an Agilent 1100 LC/MSD spectrometer using the following conditions: Capcell Pak MGⅡ column (3 *μ*m, 2.0 × 100 mm); column temperature at 30°C; mobile phase, solvent A (.1% FA in H_2_O) and solvent B (0.1% FA in CH_3_CN); flow rate, 0.5 mL/min; UV detection at 340 nm; compounds **1** and **3**, under isocratic relatio of A/B (34:66); compound **2**, under a linear gradient elution mode (5–100% B for 30 min). The retention times of the corresponding *L/D*-FDLA derivatives (*m/z* 474) for **1** and **3** were 23.6 min (*L*-*N*-Me-Phe-*L*-FDLA) and 28.7 min (*L*-*N*-Me-Phe-*D*-FDLA), respectively, while the *L* -FDLA derivatives for **1** and **3** were 23.6 min. The retention times of the corresponding *L/D*-FDLA derivatives for **2** were 5.6 min (*L*-*N*-Me-Phe-*L*-FDLA, *m/z* 474), 9.5 min (*L*-*N*-Me-Phe-*D*-FDLA, *m/z* 474), 4.6 min (*L*-Phe-*L*-FDLA, *m/z* 460), and 14.0 min (*L*-Phe-*D*-FDLA, *m/z* 460), respectively, while the *L* -FDLA derivatives for **2** were 5.6 min (*L*-*N*-Me-Phe-*L*-FDLA, *m/z* 474) and 4.6 min (*L*-Phe-*L*-FDLA, *m/z* 460), respectively. Consequently, the absolute configuration of the *N*-Me-Phe and Phe moieties in **1**–**3** were assigned as *L* (Supplementary Figures S1, S3).

### Chiral HPLC analysis of the hydrolyzate

Above the third portion of hydrolysate was performed by HPLC analysis using a ligand-exchange-type chiral column: MCI gel CRS10W, 4.6 × 50 mm, 5 *μ*m; fow rate 1 mL/min, eluent 2 mM aqueous CuSO_4_, UV detection at 254 nm (Chen et al., 2018). Standard *R*-Hiv, *S*-Hiv, *R*-HL and *S*-HL were used co-injection experiments and their retention times (*t*_R_, min) were as follows: *R*-Hiv (43.4), *S*-Hiv (69.7), *R*-HL (5.9), *S*-HL (7.8). These results of the HPLC analysis established the *R*-configuration for Hiv and HL units in **1**–**3** (Supplementary Figure S4).

### Cytotoxic activity assessment (Chen et al., 2018)

The cytotoxic effects of all compounds against human pancreatic cancer cell line (ASPC-1) were evaluated by CCK-8 method. The gemcitabine (IC_50_, 1.53 *μ*M) was used as the positive drug.

### Antiviral activity assessment (Song et al., 2022)

Briefly, the H460 cells were inoculated into 96 well culture plates and cultured at 35°C under 5% CO_2_ condition, and infected by HCoV-OC43 virus with 100 times 50% tissue culture infective dose (TCID50) 24 h later; then the positive control drugs and test compounds were added. The half inhibitory concentration (IC_50_) and the half toxic concentration (TC_50_) were determined by the Reed and Muench method. The selectivity index (SI) was calculated as the ratio of TC_50_/IC_50_. The ribavirin (IC_50_, 19.24 *μ*g/mL; SI, 5.2) was used as the positive drug.

## Conclusion

In conclusion, three undescribed hexadepsipeptides, together with six known compounds were separated from the fungus *Fusarium* sp. CPCC400857. Their structures including the absolute configuration were determined by the extensive analysis of spectroscopic data, advanced Marfey’s method, and chiral HPLC analysis. Pancreatic cancer is one of the most difficult and invasive tumors of digestive system, with low resection rate (Wang et al., 2022). But recent years, few active molecules have been found for pancreatic cancer from microorganisms. Leucinostatin Y, a peptaibiotic isolated from the entomoparasitic fungus *Purpureocillium lilacinum* selectively suppressesed the growth of human pancreatic cancer cells, including PANC-1, BxPC-3, PSN-1, and PK-8 (Momose et al., 2019). Two benzophenone derivatives, pestalones C and E were found to suppress the pancreatic cancer cell line PANC-1 with IC_50_ values of 7.6 and 7.2 μM, respectively (Wang et al., 2019). In our previous study, we have found secoemestrin C, an epipolythiodioxopiperazine compound, displayed significant cytotoxicity against several pancreatic adenocarcinoma cells, and enhanced the endoplasmic reticulum stress by a unique mechanism with downregulation of the YAP *via* the destruction complex (YAP-Axin-GSK-βTrCP) (Wang et al., 2022). Beauvericin (**4**) had shown cytotoxicity to the hepatocellular carcinoma-line Hep G2 and fibroblast-like foetal lung cell line MRC-5 in previous report (Ivanova et al., 2006). In this work, compounds **4**, and **7**–**9** exhibited moderate cytotoxicity against the human pancreatic cancer cell ASPC-1, which indicated that beauvericins and 4-hydroxy-2-pyridones may be used as the leading molecules of anti-pancreatic cancer, providing clues for pharmaceutical chemists and pharmacologists. Comparison of the cytotoxicity of **1**–**5** demonstrated that the cyclo-form and nitrogen methylation at *L*-phe residue are important for the cytotoxic activity. Compounds **7**–**9** showed more cytotoxic than **6** indicating that the 5-aromatic ring may decrease the toxicity to the ASPC-1 cell.

The global outbreak of the COVID-19 pandemic has caused serious public health and social problems. Although several drugs, such as remdesivir, molnupiravir, nirmatrelvir/ritonavir, and azvudine have been successively approved (Zhang et al., 2021; Zhang et al., 2022), the effective anti-COVID-19 drugs is still one of the major researches focuses. Here, we firstly discovered compounds **7** and **8** displayed the antiviral activity against the coronavirus (HCoV-OC43), but this type of compounds would need further structural modification, to lower toxicity and improve their values of selectivity index (SI).

## Data Availability

The original contributions presented in the study are included in the article/Supplementary Material, further inquiries can be directed to the corresponding authors.
