# Supplementary material for: Cytotoxic hexadepsipeptides and anti-coronaviral 4-hydroxy-2-pyridones from an endophytic Fusarium sp
Source: Front Chem. 2023 Jan 12;10:1106869. doi: 10.3389/fchem.2022.1106869 (PMC9877305; doi:10.3389/fchem.2022.1106869)
Supplement: Supplementary file 1 [file DataSheet1.pdf]

Cytotoxic Hexadepsipeptides and Anti-coronaviral 4-Hydroxy-2-pyridones from an Endophytic *Fusarium* sp.

Shanshan Chang [†], Biying Yan [†], Yuchuan Chen, Wuli Zhao, Rongmei Gao, Yuhuan Li, Liyan Yu, Yunying Xie, Shuyi Si *, and Minghua Chen *

Institute of Medicinal Biotechnology, Chinese Academy of Medical Sciences & Peking Union Medical College, Tiantan xili No.1, Beijing 100050, P. R. China.

Supporting Information

1. Physicochemical properties and NMR Data of 4-9.

2. The advanced Marfey's analysis of the acid hydrolysate of compounds 1-3

Figure S1. The advanced Marfey's analysis of the acid hydrolysate of **1**

Figure S2. The advanced Marfey's analysis of the acid hydrolysate of **2**

Figure S3. The advanced Marfey's analysis of the acid hydrolysate of **3**

Table S1. LC-MS Retention Times (t_R, minute) of the FDLA-Derivatized Amino Acids in Marfey's Analysis

3. Chiral HPLC analysis of the hydrolyzate of compounds 1-3

Figure S4. Chiral HPLC analysis of the hydrolyzate of **1-3**

Table S2. Chiral HPLC Retention Times (t_R, minute) of the Hiv and HL hydrolyzated from compounds **1-3**

4. Spectral information of 1

Figure S5. UV spectrum of **1** in MeOH

Figure S6. IR spectrum of **1**

Figure S7. HR-ESIMS spectrum of **1**

Figure S8. Fragments observed for **1** by HRESIMS/MS

Figure S9. ^{1}H NMR spectrum of **1** in DMSO-d_6

Figure S10. ^{13}C NMR spectrum of **1** in DMSO-d_6

Figure S11. DEPT spectrum of **1** in DMSO-d_6

Figure S12. HSQC spectrum of **1** in DMSO-d_6

Figure S13. ^{1}H-^{1}H COSY spectrum of **1** in DMSO-d_6

Figure S14. HMBC spectrum of **1** in DMSO-d_6

Figure S15. NOESY spectrum of **1** in DMSO-d_6

5. Spectral information of 2

Figure S16. UV spectrum of **2** in MeOH

Figure S17. IR spectrum of **2**

Figure S18. HR-ESIMS spectrum of **2**

Figure S19. Fragments observed for **2** by HRESIMS/MS

Figure S20. ^{1}H NMR spectrum of **2** in DMSO-d_6

Figure S21. ^{13}C NMR spectrum of **2** in DMSO-d_6

Figure S22. DEPT spectrum of **2** in DMSO-d_6

Figure S23. HSQC spectrum of **2** in DMSO-d_6

Figure S24. ^{1}H-^{1}H COSY spectrum of **2** in DMSO-d_6

Figure S25. HMBC spectrum of **2** in DMSO-d_6

Figure S26. NOESY spectrum of **2** in DMSO-d_6

6. Spectral information of 3

Figure S27. UV spectrum of **3** in MeOH

Figure S28. IR spectrum of **3**

Figure S29. HR-ESIMS spectrum of **3**

Figure S30. Fragments observed for **3** by HRESIMS

Figure S31. ^{1}H NMR spectrum of **3** in DMSO-d_6

Figure S32. ^{13}C NMR spectrum of **3** in DMSO-d_6

Figure S33. DEPT spectrum of **3** in DMSO-d_6

Figure S34. HSQC spectrum of **3** in DMSO-d_6

Figure S35. ^{1}H-^{1}H COSY spectrum of **3** in DMSO-d_6

Figure S36. HMBC spectrum of **3** in DMSO-d_6

Figure S37. NOESY spectrum of **3** in DMSO-d_6

1. Physicochemical properties and NMR Data of 4-9

Beauvericin (**4**): White amorphous powder; $[\alpha]_D^{20}$ +15.6 (*c* 0.3, MeOH); UV (LC) 217 nm; ^{1}H NMR (600 MHz, DMSO-d_6): δ_H 7.26-7.15 (15H, m, H-5~H-9, H-19~H-23, H-33~H-37), 5.42 (3H, dd, *J* = 4.8, 12.0 Hz, H-2, H-16, H-30), 4.82 (3H, d, *J* = 9.0 Hz, H-11, H-25, H-39), 3.17 (3H, d, *J* = 4.8, 14.4 Hz, H-3a, H-17a, H-31a), 3.04 (3H, d, *J* = 12.0, 14.4 Hz, H-3b, H-17b, H-31b), 3.01 (9H, s, *N*-CH_3-10, *N*-CH_3-24, *N*-CH_3-38), 1.76 (3H, m, H-12, H-26, H-40), 0.74 (9H, d, *J* = 6.0 Hz, H-13, H-27, H-41), 0.23 (9H, d, *J* = 7.2 Hz, H-14, H-28, H-42); ^{13}C NMR (150 MHz, DMSO-d_6): δ_C 16.4 (C-13, C-27, C-41), 18.1 (C-14, C-28, C-42), 29.5 (C-12, C-26, C-40), 31.3 (*N*-CH_3-10, *N*-CH_3-24, *N*-CH_3-38), 34.0 (C-3, C-17, C-31), 55.7 (C-2, C-16, C-30), 74.6 (C-11, C-25, C-39), 126.5 (C-7, C-21, C-35), 128.3 (C-5, C-9, C-19, C-23, C-33, C-37), 128.7 (C-6, C-8, C-20, C-22, C-34, C-36), 136.7 (C-4, C-18, C-32), 169.2 (C-10, C-24, C-38), 169.3 (C-1, C-15, C-29).

Beauvericin D (**5**): White amorphous powder; $[\alpha]_D^{20}$ +9.8 (*c* 0.1, MeOH); UV (LC) 217 nm; ^{1}H NMR (600 MHz, DMSO-d_6): δ_H 4.85 (1H, d, *J* = 7.8 Hz, H-11), 4.90 (1H, d, *J* = 9.0 Hz, H-39), 4.67 (1H, d, *J* = 8.4 Hz, H-25), 1.76 (H, m, H-12), 1.87 (H, m, H-40), 1.76 (1H, m, H-26), 0.60 (3H, d, *J* = 6.6 Hz, H-13), 0.19 (3H, d, *J* = 6.6 Hz, H-41), 0.74 (3H, d, *J* = 6.6 Hz, H-28), 0.36 (3H, d, *J* = 6.6 Hz, H-14), 0.53 (3H, d, *J* = 6.6 Hz, H-42), 0.73 (3H, d, *J* = 6.6 Hz, H-27), 2.94 (3H, s, *N*-CH_3-10), 2.99 (3H, s, *N*-CH_3-38), 8.22 (1H, d, *J* = 8.4 Hz, NH-24), 5.52 (1H, dd, *J* = 4.6, 12.0 Hz, H-2), 5.29 (1H, m, H-30), 4.62 (1H, m, H-16), 3.18 (1H, dd, *J* = 4.8, 14.4 Hz, H-3a), 2.98 (1H, dd, *J* = 5.4, 14.4 Hz, H-3b), 3.35 (1H, dd, *J* = 4.8, 10.8 Hz, H-31a), 3.27 (1H, dd, *J* = 5.4, 10.8 Hz, H-31b), 3.04 (1H, dd, *J* = 4.8, 14.4 Hz, H-17a), 3.18 (1H, dd, *J* = 4.8, 14.4 Hz, H-17b), 7.01-7.26 (15H, m, H-5~H-9, H-19~H-23, H-33~H-37); ^{13}C NMR (150 MHz, DMSO-d_6): δ_C 74.8 (C-11), 74.5(C-39), 78.4 (C-25), 29.3 (C-12), 29.0 (C-40), 29.2 (C-26), 17.7 (C-13), 16.4 (C-41), 16.9 (C-28), 17.8 (C-14), 17.7 (C-42), 18.0 (C-27), 170.5 (C-10), 169.4 (C-38), 169.2 (C-24), 32.0 (*N*-CH_3-10), 30.9 (*N*-CH_3-38), 63.0 (C-2), 55.5 (C-30), 52.3 (C-16), 33.9 (C-3), 33.4 (C-31), 37.4 (C-17), 136.7 (C-4), 136.8 (C-32), 136.6 (C-18), 126.5-129.1 (C-5~C-9, C-19~C-23, C-33~C-37), 169.1 (C-1), 167.3 (C-29), 169.1 (C-15).

(–)-Sambutoxin (**6**): White amorphous powder; $[\alpha]_D^{20}$ –98.7 (*c* 0.3, MeOH); UV (LC) 219, 296 nm; ^{1}H NMR (600 MHz, DMSO-d_6): δ_H 9.69 (1H, s, OH-4), 7.55 (1H, s, H-6), 4.83 (1H, dd, *J* = 1.8, 11.4 Hz, H-7), 1.49 (1H, m, H-8a), 1.98 (1H, m, H-8b), 1.34 (1H, m, H-9a), 1.85 (1H, m, H-9b), 1.53 (1H, m, H-10), 3.27 (1H, dd, *J* = 4.2, 10.8 Hz, H-11), 5.20 (1H, d, *J* = 9.6 Hz, H-13), 2.27 (1H, t, *J* = 7.2 Hz, H-14),1.01 (1H, m, H-15a), 1.18 (1H, m, H-15b), 1.23 (1H, m, H-16), 1.01 (1H, m, H-17a), 1.22 (1H, m, H-17b), 0.79 (3H, t, *J* = 7.2 Hz, H-18), 0.80 (3H, d, *J* = 6.0 Hz, H-19), 0.86 (3H, d, *J* = 6.6 Hz, H-20), 1.56 (3H,s, H-21), 0.69 (3H, d, *J* = 6.6 Hz, H-22), 3.37 (3H, s, H-23), 7.19 (1H, d, *J* = 8.4 Hz, H-2′), 6.75 (1H, d, *J* = 8.4 Hz, H-3′), 9.41 (1H, s, OH-4′), 6.75 (1H, d, *J* = 8.4 Hz, H-5′), 7.19 (1H, d, *J* = 8.4 Hz, H-6′); ^{13}C NMR (150 MHz, DMSO-d_6): δ_C 160.2 (C-2), 108.7 (C-3), 160.9 (C-4), 112.8 (C-5), 136.9 (C-6), 76.9 (C-7), 30.2 (C-8), 31.5 (C-9), 31.6 (C-10), 91.2 (C-11), 130.4 (C-12), 137.1 (C-13), 29.1 (C-14), 44.3 (C-15), 31.5 (C-16), 28.0 (C-17), 11.0 (C-18), 19.6 (C-19), 20.8 (C-20), 11.3 (C-21), 17.3 (C-22), 36.1 (C-23), 124.5 (C-1′), 130.1 (C-2′), 114.9 (C-3′), 156.5 (C-4′), 114.9 (C-5′), 130.1 (C-6′).

(–)-Oxysporidinone (**7**): White amorphous powder; $[\alpha]_D^{20}$ –51.2 (*c* 0.9, MeOH); UV (LC) 219, 296 nm; ^{1}H NMR (600 MHz, DMSO-d_6): δ_H 9.88 (1H, s, OH-4), 7.58 (1H, s, H-6), 4.32 (1H, dd, *J* = 5.4, 9.6 Hz, H-7), 1.54 (1H, m, H-8a), 2.00 (1H, m, H-8b), 1.33 (1H, m, H-9a), 1.82 (1H, m, H-9b), 1.66 (1H, m, H-10), 3.52 (1H, d, *J* = 9.6 Hz, H-11), 5.18 (1H, dd, *J* = 1.8, 9.6 Hz, H-13), 2.38

(1H, m, H-14), 1.00 (1H, m, H-15a), 1.18 (1H, m, H-15b), 1.31 (1H, m, H-16), 1.00 (2H, m, H-17), 0.79 (3H, t, *J* = 6.6 Hz, H-18), 0.69 (3H, d, *J* = 6.6 Hz, H-19), 1.57 (3H, d, *J* = 1.8 Hz, H-20), 0.87 (3H, d, *J* = 6.6 Hz, H-21), 0.79 (3H, d, *J* = 6.6 Hz, H-22), 3.33 (3H, s, H-23), 2.47 (1H, m, H-2′a), 2.00 (1H, m, H-2'b), 2.28 (1H, dd, *J* = 5.4, 13.8 Hz, H-3′a), 2.38 (1H, dd, *J* = 5.4, 13.8 Hz, H-3′b), 2.69 (1H, dd, J = 13.8, 11.4 Hz, H-5′a), 2.63 (1H, dt, *J* = 13.8, 6.6 Hz, H-5′b), 4.89 (1H, dd, *J* = 13.8, 6.6 Hz, H-6′), 9.88 (1H, OH-4); ^{13}C NMR (150 MHz, DMSO-d_6): δ_C 160.5 (C-2), 108.7 (C-3), 161.0 (C-4), 114.7 (C-5), 136.5 (C-6), 73.3 (C-7), 29.0 (C-8), 31.6 (C-9), 31.5 (C-10), 91.0 (C-11), 130.6 (C-12), 136.8 (C-13), 29.9 (C-14), 46.4 (C-15), 31.5 (C-16), 28.0 (C-17), 11.8 (C-18), 17.3 (C-19), 11.0 (C-20), 20.8 (C-21), 19.6 (C-22), 36.5 (C-23), 77.0 (C-1′), 31.8 (C-2′), 36.1 (C-3′), 209.1 (C-4′), 44.4 (C-5′), 69.9 (C-6′).

Fusapyridon A (**8**): White amorphous powder; $[\alpha]_D^{20}$ –48.6 (*c* 0.8, MeOH); UV (LC) 219, 296 nm; ^{1}H NMR (600 MHz, DMSO-d_6): δ_H 7.73 (1H, s, H-6), 4.54 (1H, dd, *J* = 2.4, 11.4 Hz, H-7), 2.11 (1H, m, H-8a), 1.34 (1H, m, H-8b), 1.77 (1H, m, H-9a), 1.18 (1H, m, H-9b), 1.45 (1H, m, H-10), 3.20 (1H, d, *J* = 16.2 Hz, H-11), 5.00 (1H, dd, *J* = 11.2, 9.0 Hz, H-13), 2.41(1H, m, H-14), 1.16 (1H, m, H-15a), 1.00 (1H, m, H-15b), 1.28 (1H, m, H-16), 1.29 (1H, m, H-17a), 1.00 (1H, m, H-17b), 0.81 (3H, t, *J* = 6.6 Hz, H-18), 0.64 (3H, d, *J* = 6.6 Hz, H-19), 1.46 (3H, d, *J* = 1.2 Hz, H-20), 0.84 (3H, d, *J* = 6.6 Hz, H-21), 0.79 (3H, d, J = 6.6 Hz, H-22), 3.34 (3H, s, H-23), 2.29 (1H, m, H-2′a), 2.21 (1H, m, H-2′b), 2.27 (1H, m, H-3′a), 1.74 (1H, m, H-3′b), 2.56 (1H, dd, *J* = 16.2, 4.2 Hz, H-5′a), 3.00 (1H, dd, *J* = 16.8, 4.8 Hz, H-5′b) 4.85 (1H, t, *J* = 4.2 Hz, H-6′), 5.97 (1H, s, OH-1′); ^{13}C NMR (150 MHz, DMSO-d_6): δ_C 162.2 (C-2), 106.8 (C-3), 165.7 (C-4), 115.5 (C-5), 134.2 (C-6), 71.2 (C-7), 28.5 (C-8), 32.5 (C-9), 31.4 (C-10), 90.1 (C-11), 134.2 (C-12), 134.2 (C-13), 28.9 (C-14), 44.5 (C-15), 31.2 (C-16), 28.1 (C-17), 11.0 (C-18), 17.6 (C-19), 10.7 (C-20), 20.9 (C-21), 19.5 (C-22), 37.1 (C-23), 75.3 (C-1′), 32.6 (C-2′), 34.7 (C-3′), 207.7 (C-4′), 42.2 (C-5′), 90.1 (C-6′).

(–)-Fusoxypyridone (**9**): White amorphous powder; $[\alpha]_D^{20}$ –22.5 (*c* 0.4, MeOH); UV (LC) 219, 296 nm; ^{1}H NMR (600 MHz, DMSO-d_6): δ_H 7.71 (1H, s, H-6), 4.57 (1H, dd, *J* = 12.0, 2.4 Hz, H-7), 1.46 (1H, m, H-8a), 1.98 (1H, m, H-8b), 1.33 (1H, m, H-9a), 1.85 (1H, m, H-9b), 1.46 (1H, m, H-10), 3.21 (1H, d, *J* = 9.6 Hz, H-11), 5.02 (1H, dd, *J* = 9.6, 1.2 Hz, H-13), 2.42 (1H, m, H-14), 1.01 (1H, m, H-15a), 1.28 (1H, m, H-15b), 1.38 (1H, m, H-16), 1.38 (1H, m, H-17), 0.80 (3H, d, *J* = 6.6 Hz, H-18), 0.65 (3H, d, *J* = 6.6 Hz, H-19), 1.48 (3H, d, *J* = 1.2 Hz, H-20), 0.87 (3H, d, *J* = 6.6 Hz, H-21), 0.78 (3H, d, *J* = 7.2 Hz, H-22), 3.33 (3H, s, H-23), 5.31 (1H, t, *J* = 4.8 Hz, H-2′), 2.53 (1H, dd, *J* = 4.8 Hz, 16.2 Hz, H-3′a), 3.00 (1H, dd, *J* = 5.4, 16.2 Hz, H-3′b), 2.37 (1H, m, H-5′a), 2.07 (1H, m, H-5′b), 2.28 (1H, m, H-6′a), 2.22 (1H, m, H-6′b), 3.10 (3H, s, OMe-1′); ^{13}C NMR (150 MHz, DMSO-d_6): δ_C 162.3 (C-2), 107.1 (C-3), 166.1 (C-4), 109.5 (C-5), 134.3 (C-6), 71.1 (C-7), 28.6 (C-8), 32.4 (C-9), 31.2 (C-10), 90.1 (C-11), 132.6 (C-12), 135.8 (C-13), 28.9 (C-14), 44.5 (C-15), 31.3 (C-16), 29.1 (C-17), 11.0 (C-18), 17.6 (C-19), 10.7 (C-20), 20.9 (C-21), 19.5 (C-22), 37.3 (C-23), 81.7 (C-1′), 85.9 (C-2′), 42.9 (C-3′), 207.5 (C-4′), 34.7 (C-5′), 31.4 (C-6′), 50.1 (OMe-1′).

2. The advanced Marfey's analysis of the acid hydrolysate of compounds 1-3

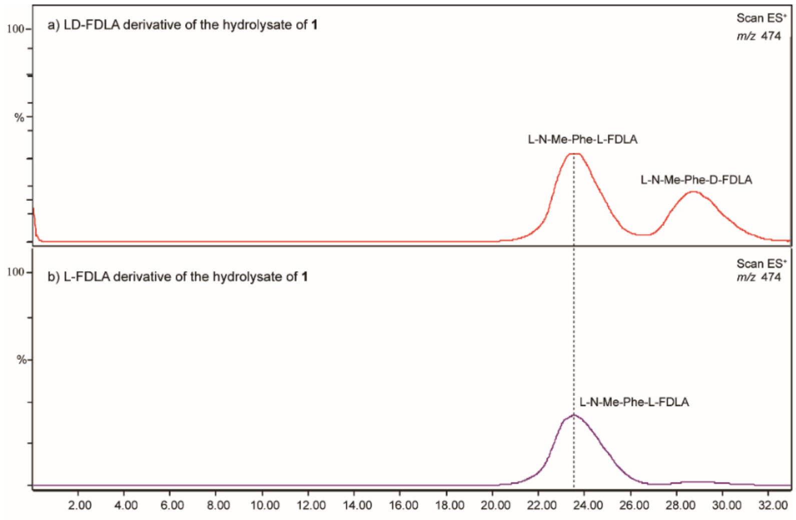

Figure S1. The advanced Marfey's analysis of the acid hydrolysate of **1**. Extracted ion chromatogram at *m/z* 474 for the FDLA derivative of *N*-Me-Phe.

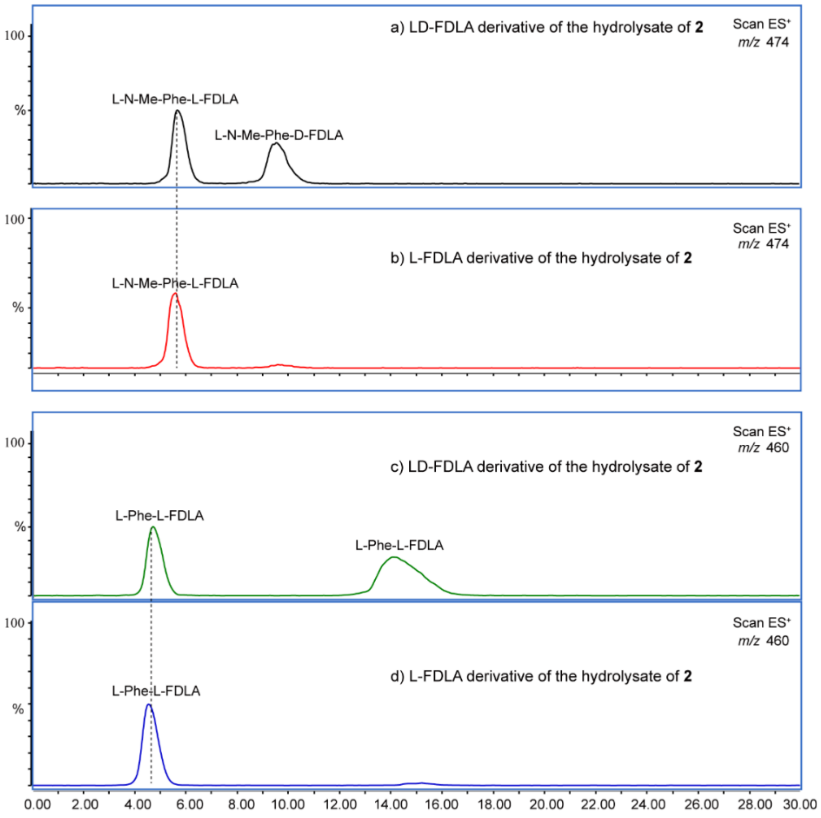

Figure S2. The advanced Marfey's analysis of the acid hydrolysate of **2**. Extracted ion chromatogram at *m/z* 474 and *m/z* 460 for the FDLA derivative of *N*-Me-Phe and Phe.

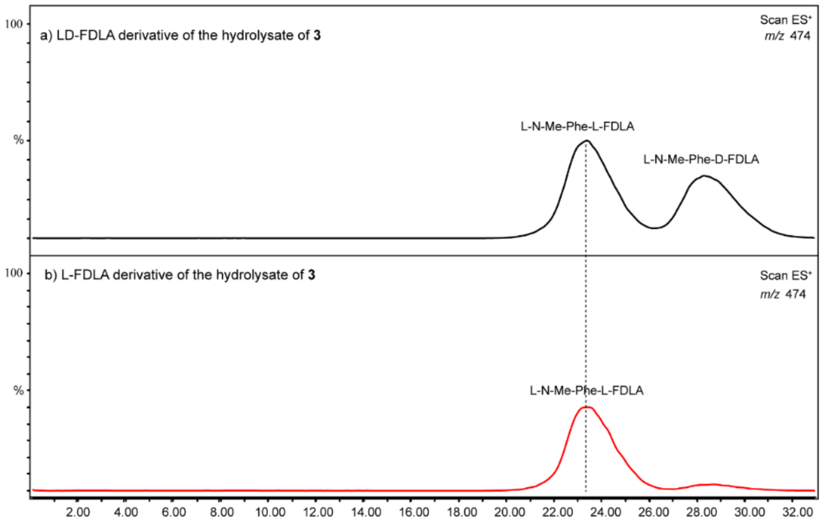

Figure S3. The advanced Marfey's analysis of the acid hydrolysate of **3**. Extracted ion chromatogram at *m/z* 474 for the FDLA derivative of *N*-Me-Phe.

Table S1. LC-MS Retention Times (t_R, min) of the FDLA-Derivatized Amino Acids in Marfey's Analysis

amino acids	*m/z* $[M+H]^+$	*L*-FDLA derivative	*D*-FDLA derivative
L-*N*-Me-Phe from **1**	474	23.6[a]	28.7[a]
L-*N*-Me-Phe from **2**	474	5.6[b]	9.5[b]
L-Phe from **2**	460	4.7[b]	14.0[b]
L-*N*-Me-Phe from **3**	474	23.6[a]	28.7[a]

[a] A (0.1% FA in H_2O) and B (0.1% FA in CH_3CN), 34:66, flow rate 0.5 mL/min, column temperature at 30 °C

[b] A (0.1% FA in H_2O) and B (0.1% FA in CH_3CN), under a linear gradient elution mode (5−100% B for 30 min) at flow rate 0.5 mL/min, column temperature at 30 °C.

3. Chiral HPLC analysis of the hydrolyzate of compounds 1-3

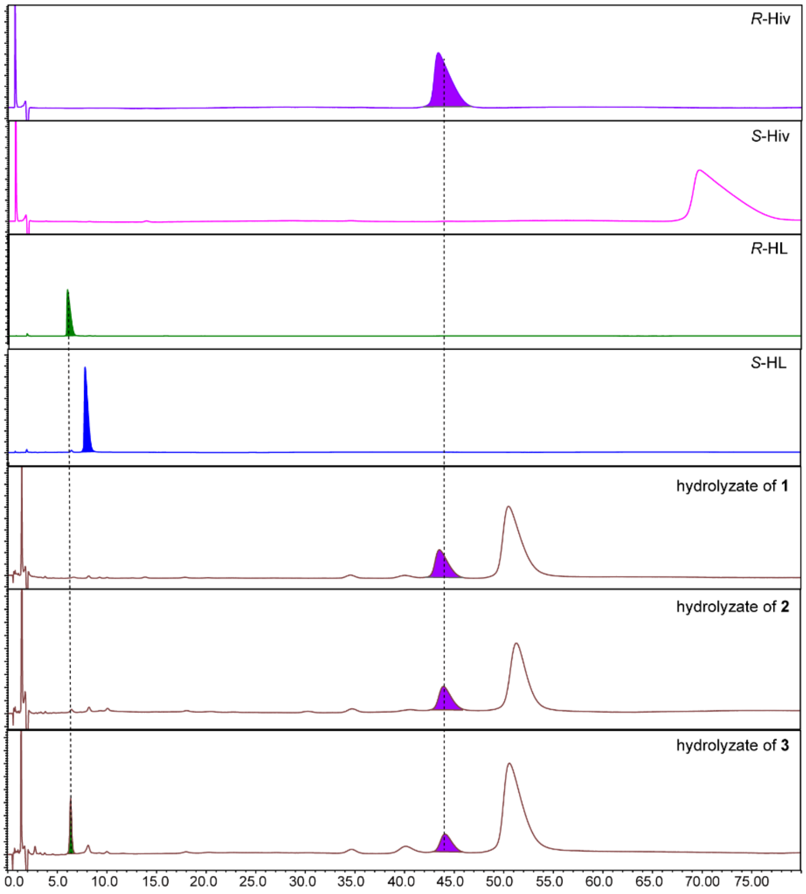

Figure S4. Chiral HPLC analysis of the hydrolyzate of **1-3**.

Table S2. The retention times (t_R, min) of the Hiv and HL hydrolyzated from compounds **1-3** by the chiral HPLC.

α-Hydroxy acid	t_R	**1**	**2**	**3**
R-Hiv	43.4	43.5	43.6	43.6
S-Hiv	69.7	-	-	-
R-HL	5.9	-	-	6.0
S-HL	7.8	-	-	-

4. Spectral information of 1

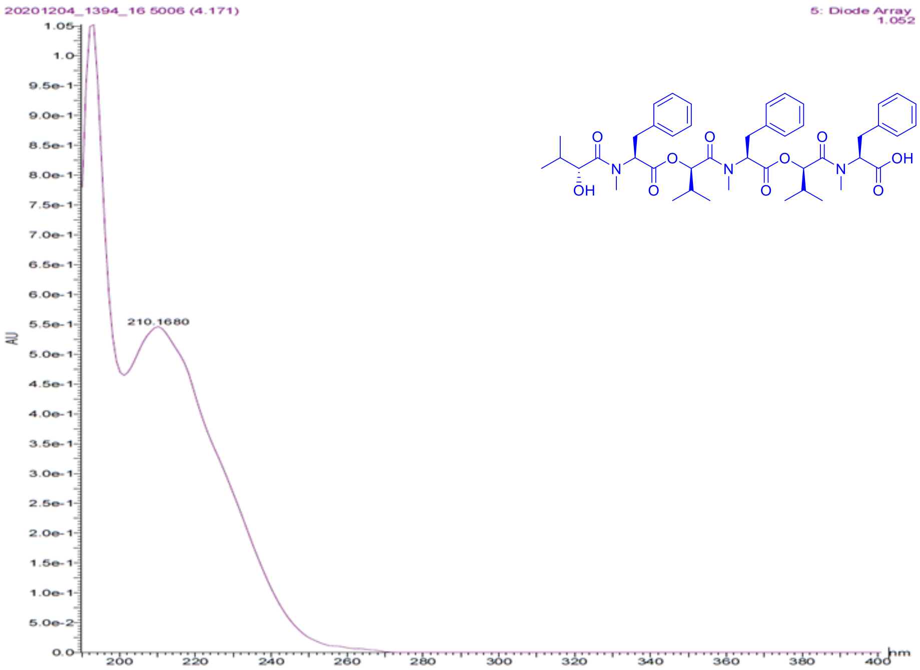

Figure S5. UV spectrum of **1** in MeOH.

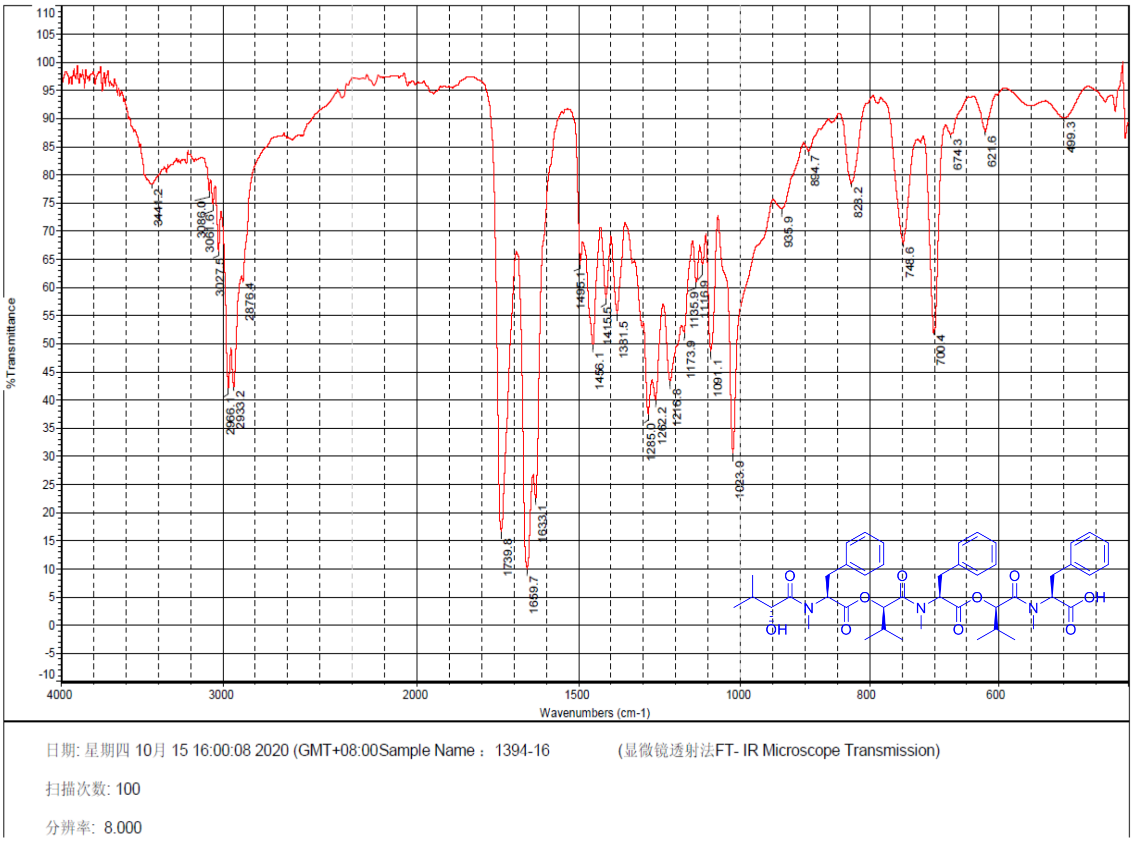

Figure S6. IR spectrum of **1**.

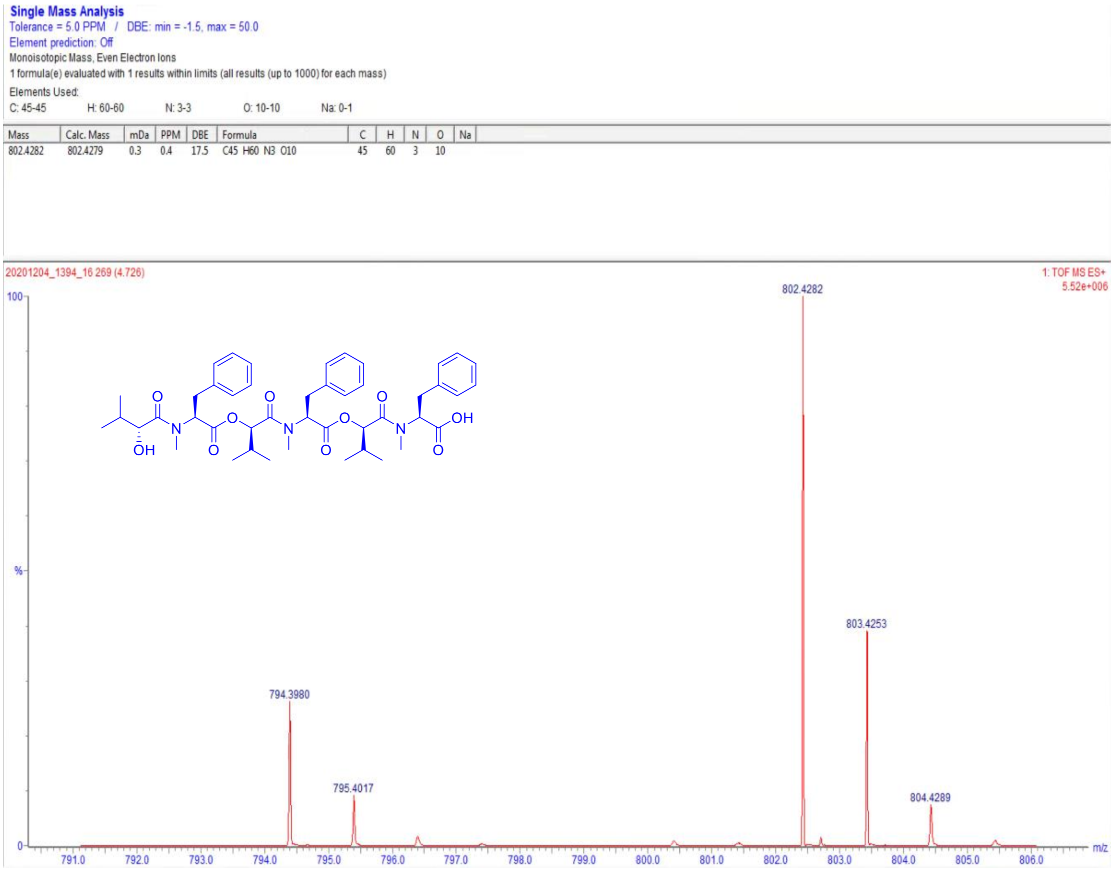

Figure S7. HR-ESIMS spectrum of **1**.

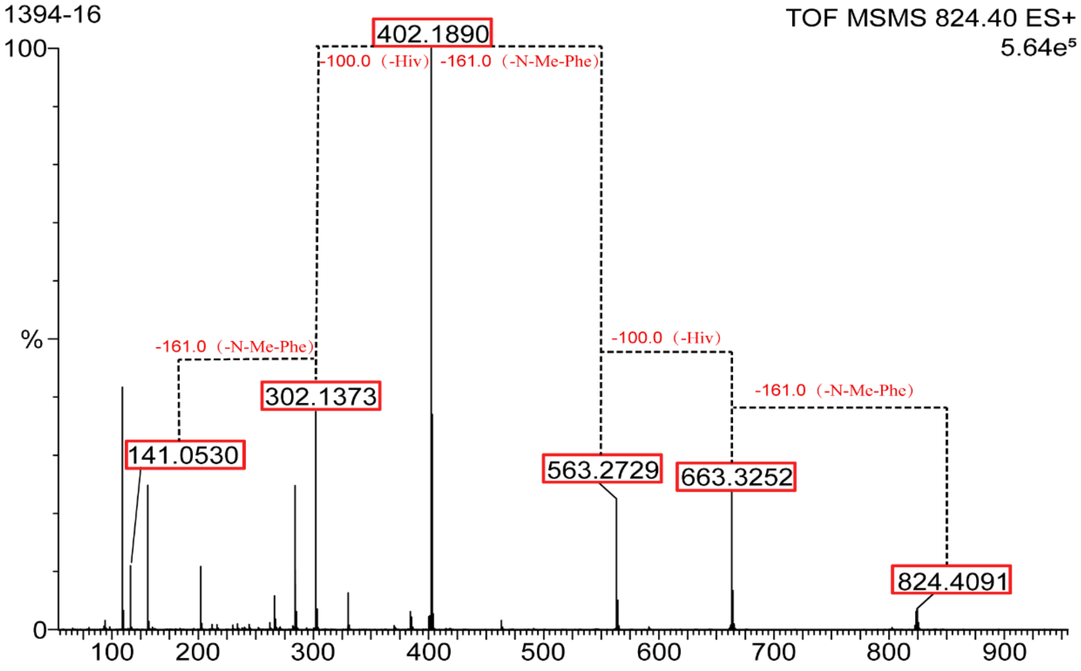

Figure S8. Fragments observed for **1** by HRESIMS/MS

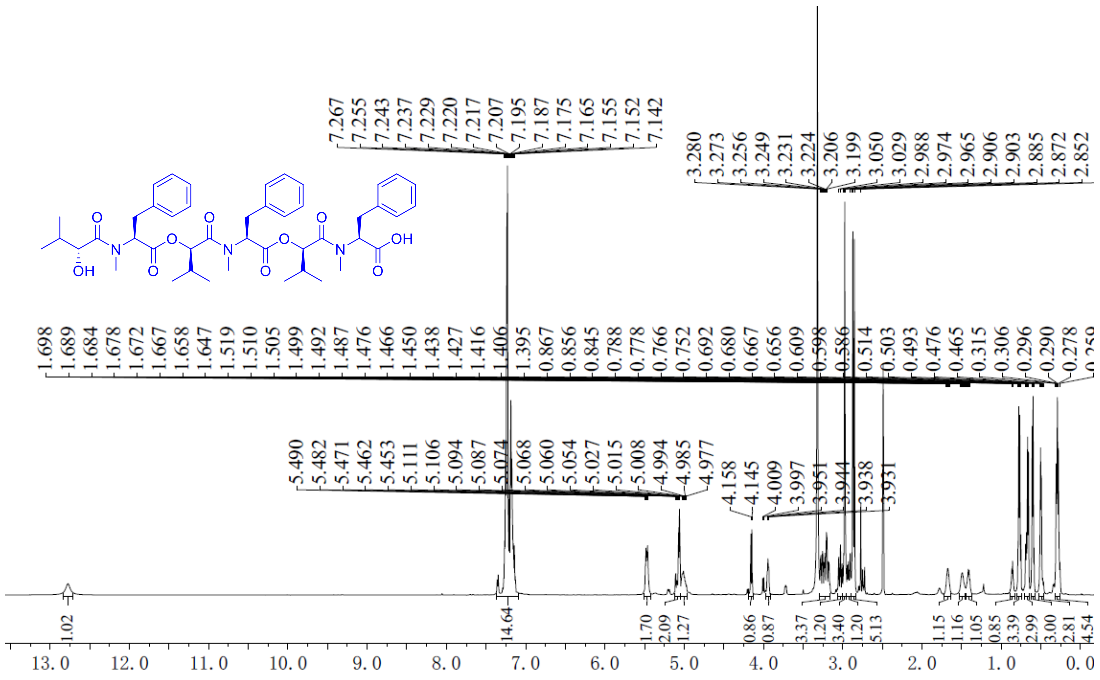

Figure S9. ^{1}H NMR (600 MHz, DMSO-d_6) spectrum of **1**.

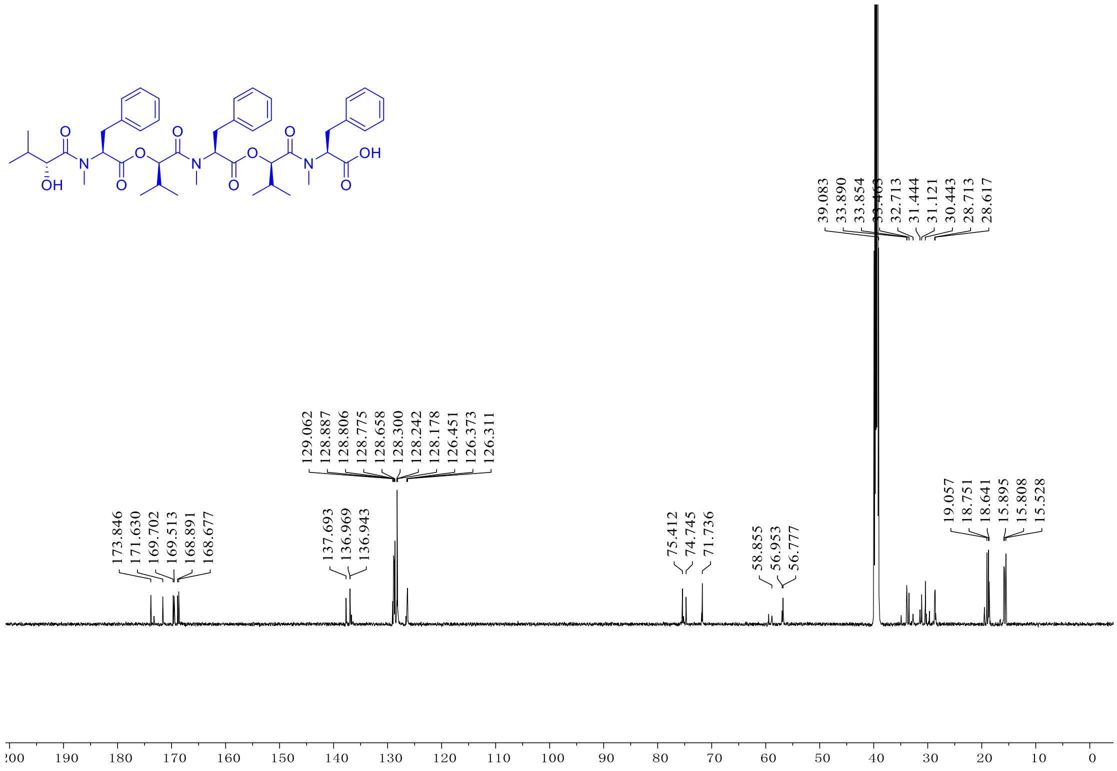

Figure S10. ^{13}C NMR (150 MHz, DMSO-d_6) spectrum of **1**.

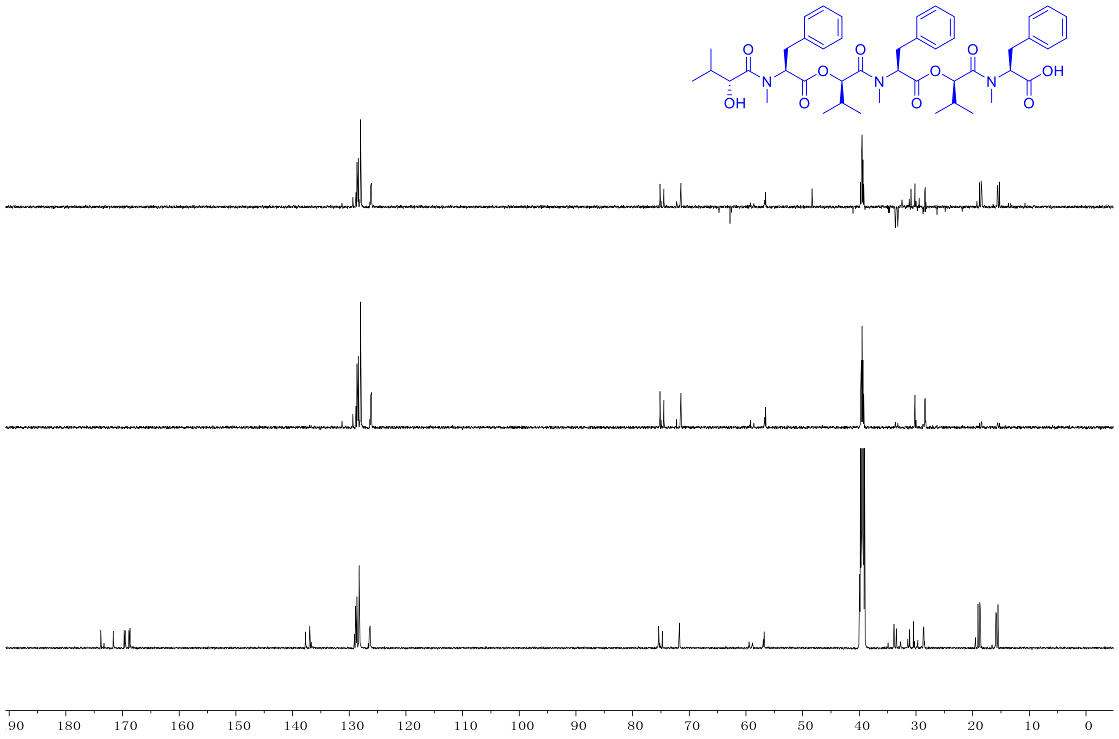

Figure S11. DEPT (150 MHz, DMSO-d_6) spectrum of **1**.

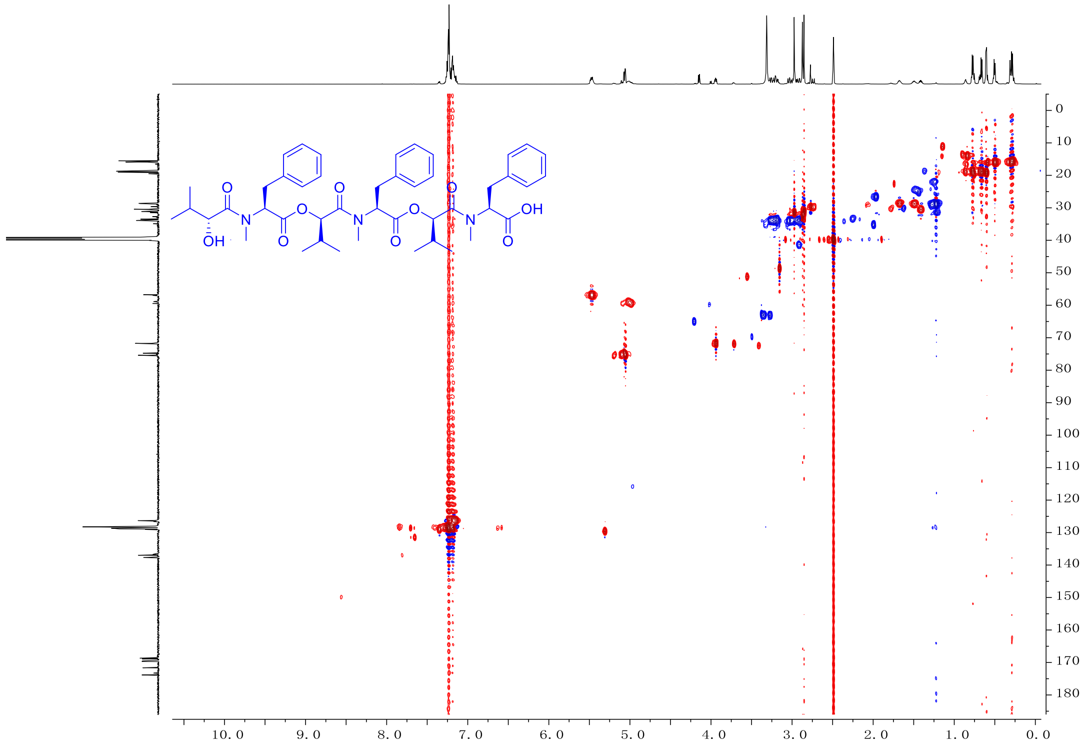

Figure S12. HSQC (600 MHz, DMSO-d_6) spectrum of **1**.

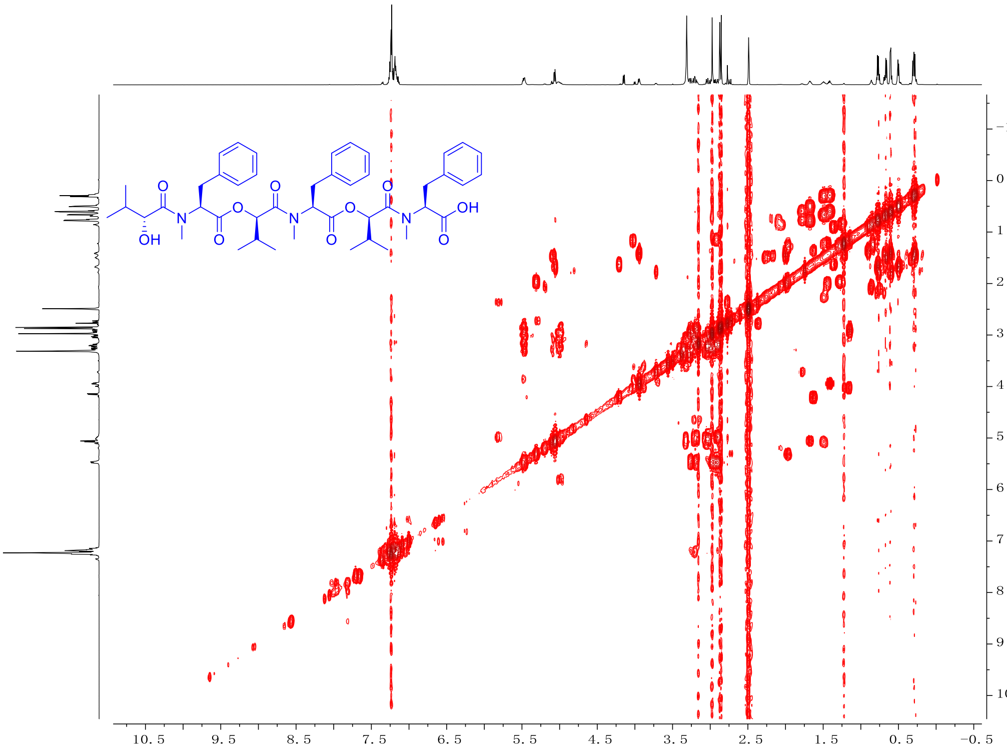

Figure S13. ^{1}H-^{1}H COSY (600 MHz, DMSO-d_6) spectrum of **1**.

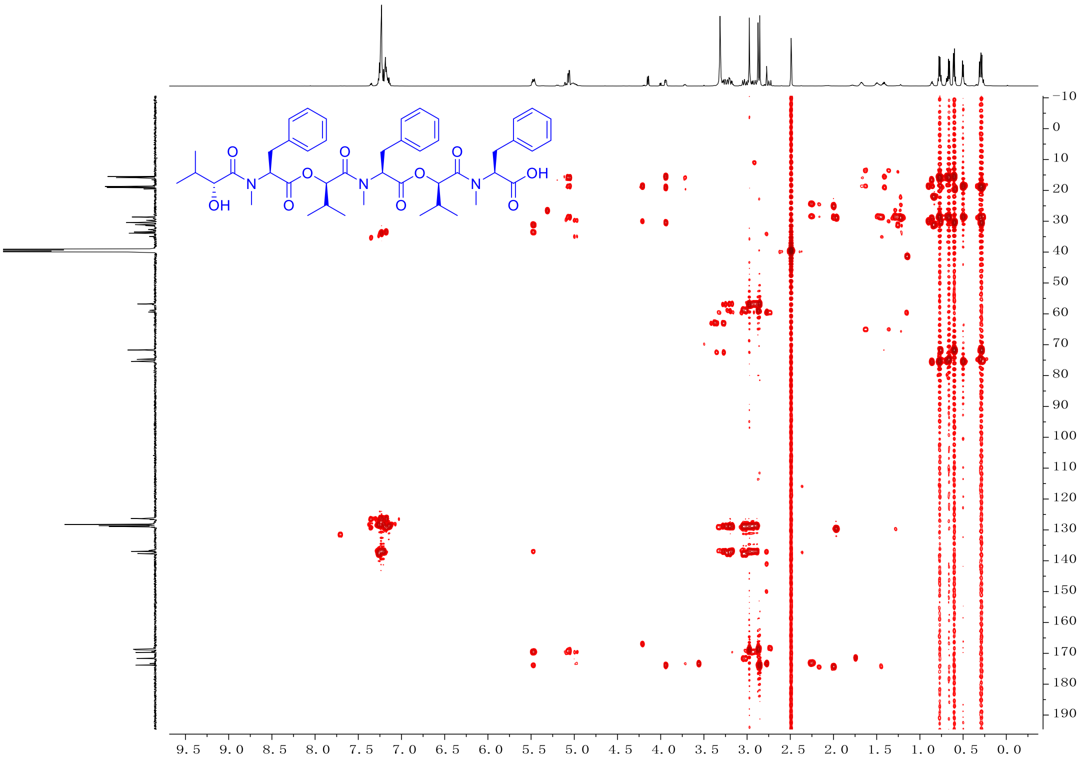

Figure S14. HMBC (600 MHz, DMSO-d_6) spectrum of **1.**

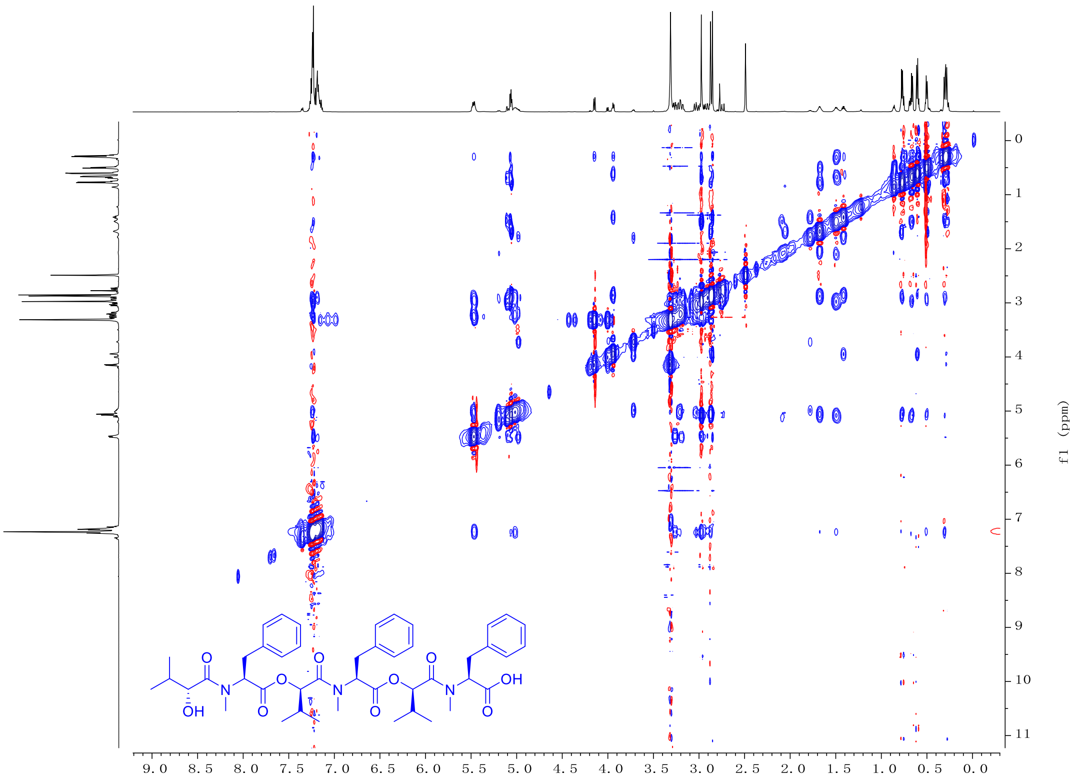

Figure S15. NOESY (600 MHz, DMSO-d_6) spectrum of **1**.

5. **Spectral information of 2**

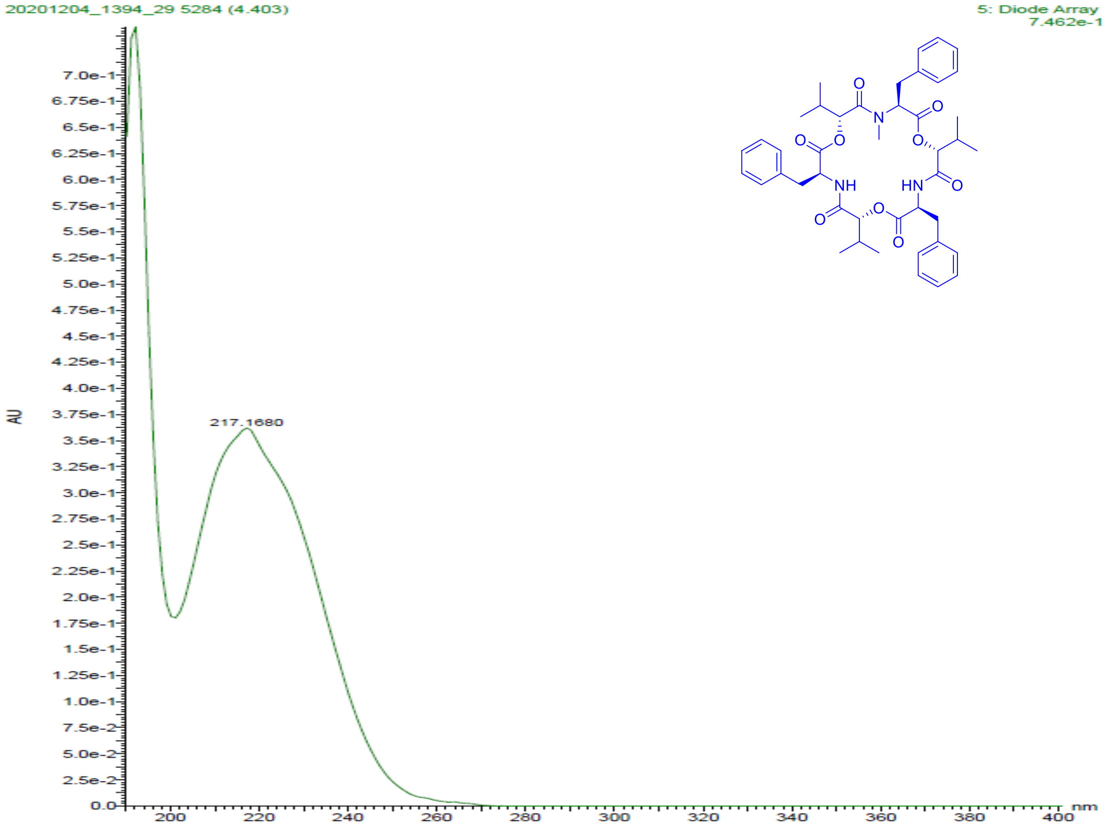

Figure S16. UV spectrum of **2** in MeOH.

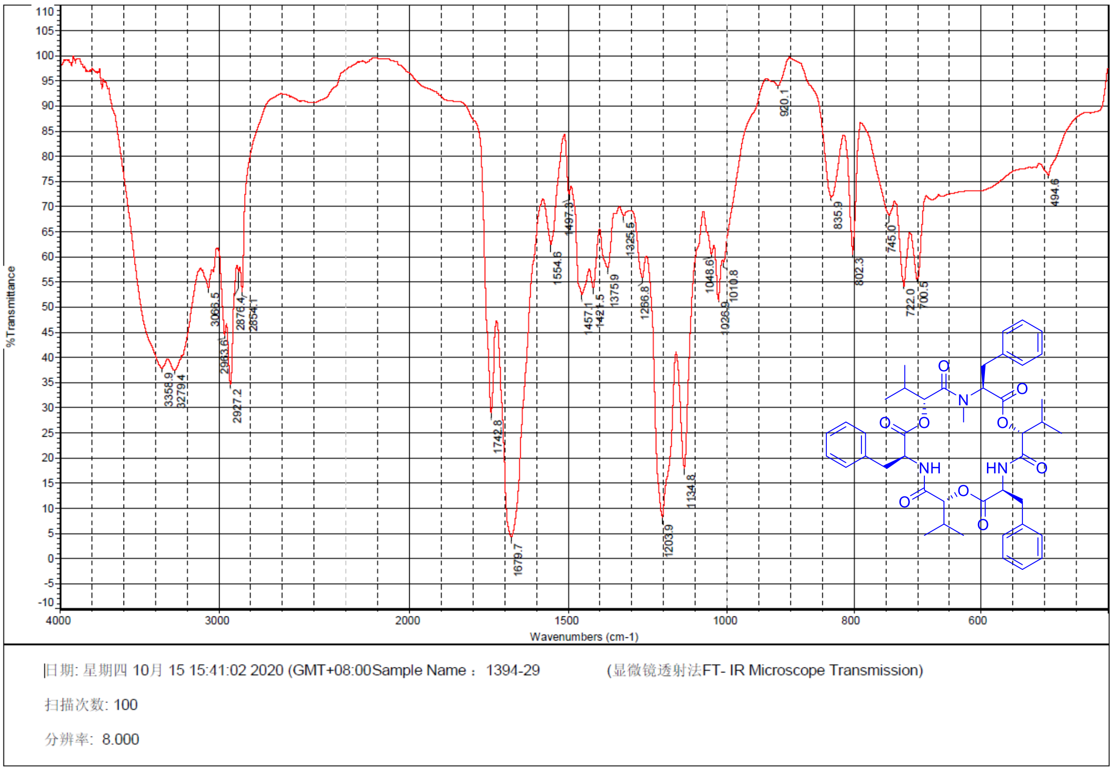

Figure S17. IR spectrum of **2**.

Single Mass Analysis
Tolerance = 5.0 PPM / DBE: min = -1.5, max = 50.0
Element prediction: Off
Monoisotopic Mass, Even Electron Ions
1 formula(e) evaluated with 1 results within limits (all results (up to 1000) for each mass)
Elements Used:
C: 43-43 H: 54-54 N: 3-3 O: 9-9 Na: 0-1

Mass	Calc. Mass	mDa	PPM	DBE	Formula	C	H	N	O	Na
756.3886	756.3860	2.6	3.4	18.5	C43 H54 N3 O9	43	54	3	9	

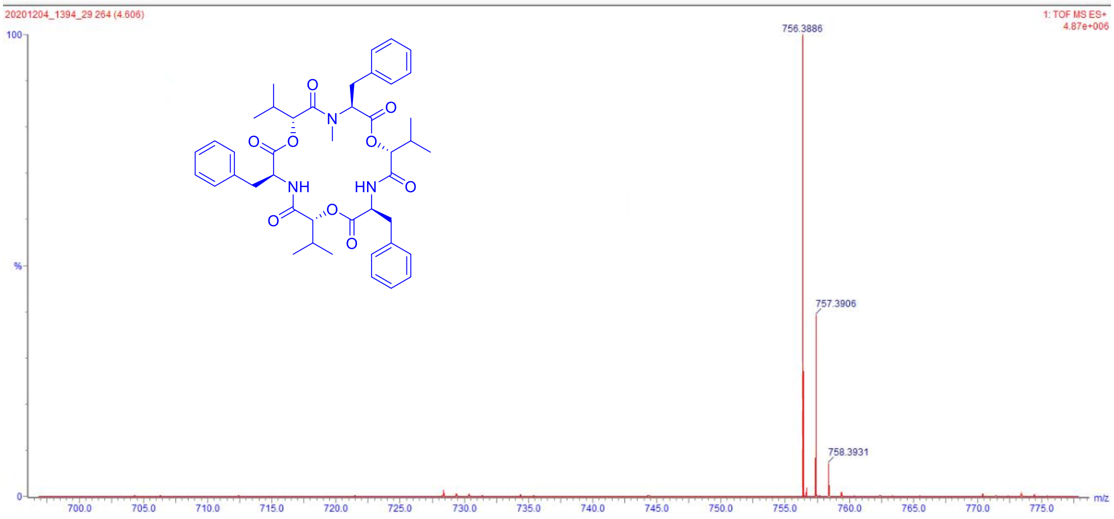

Figure S18. HR-ESIMS spectrum of **2**.

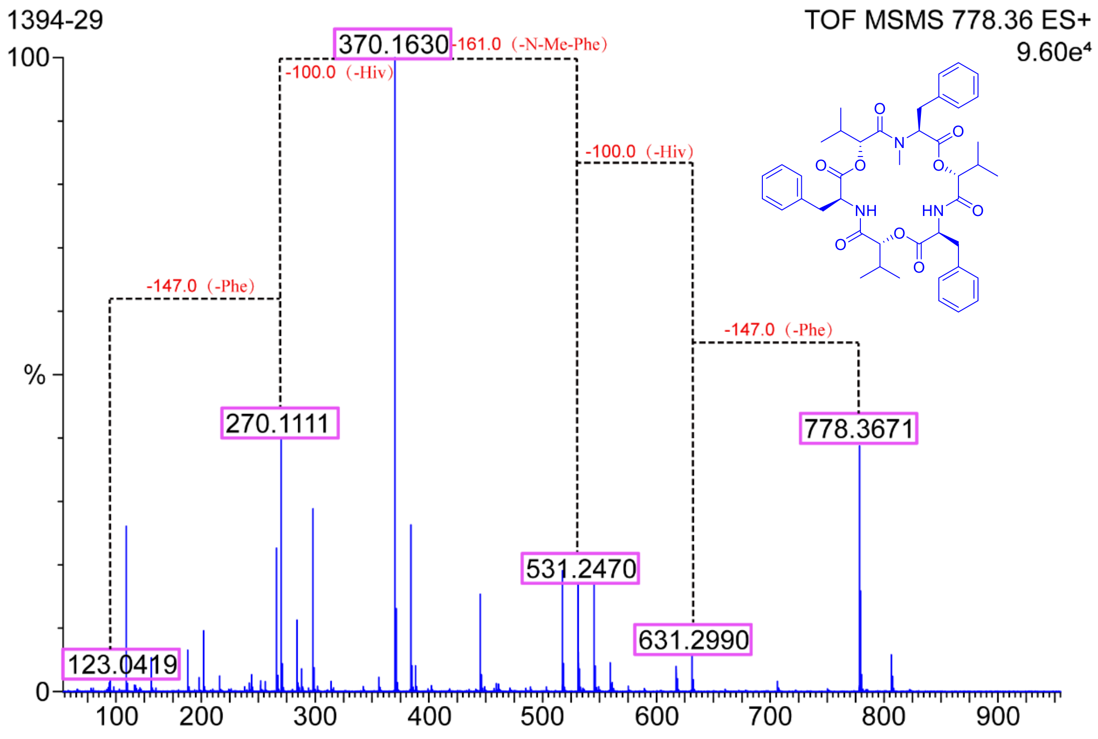

Figure S19. Fragments observed for **2** by HRESIMS/MS.

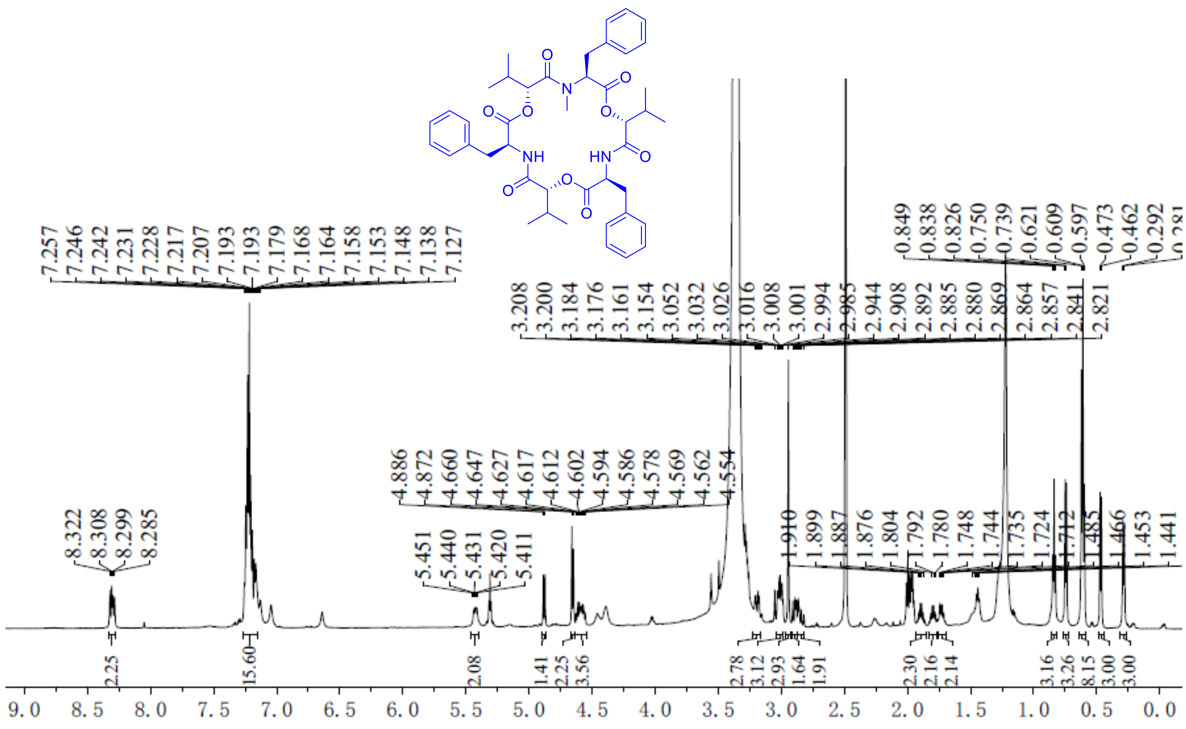

Figure S20. ^{1}H NMR (600 MHz, DMSO-d_6) spectrum of **2**.

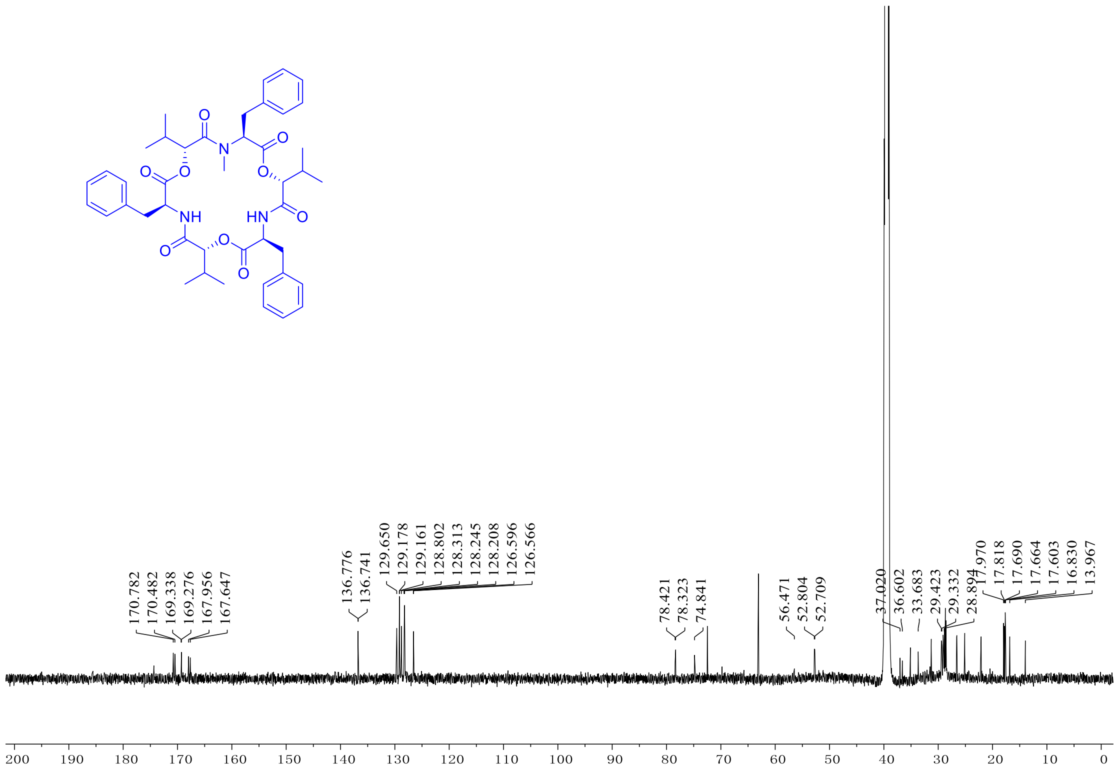

Figure S21 ^{13}C NMR (150 MHz, DMSO-d_6) spectrum of **2**.

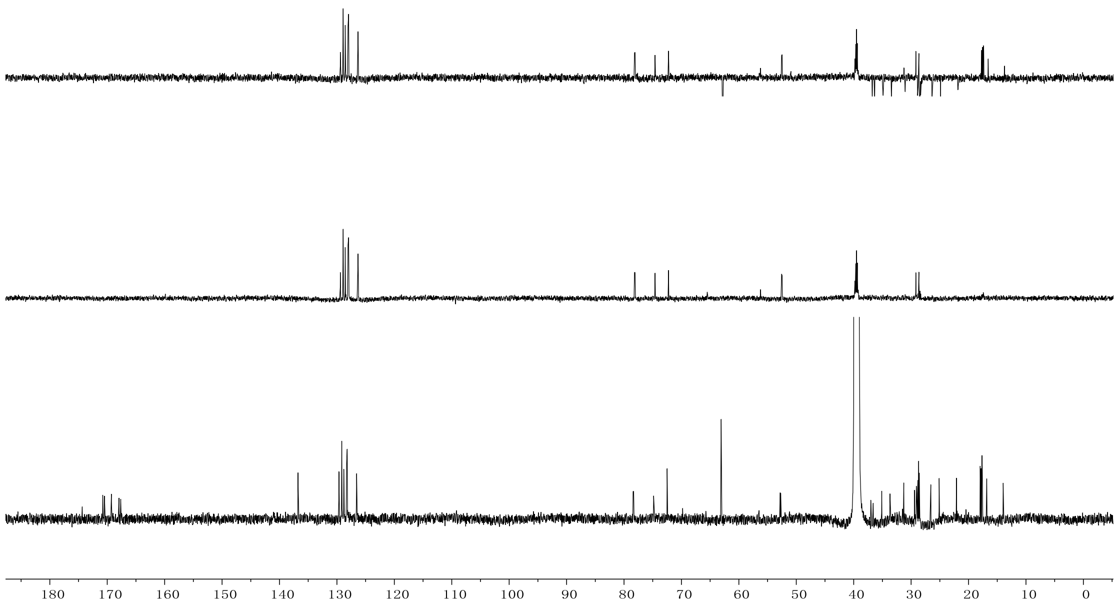

Figure S22. DEPT (150 MHz, DMSO-d_6) spectrum of **2**.

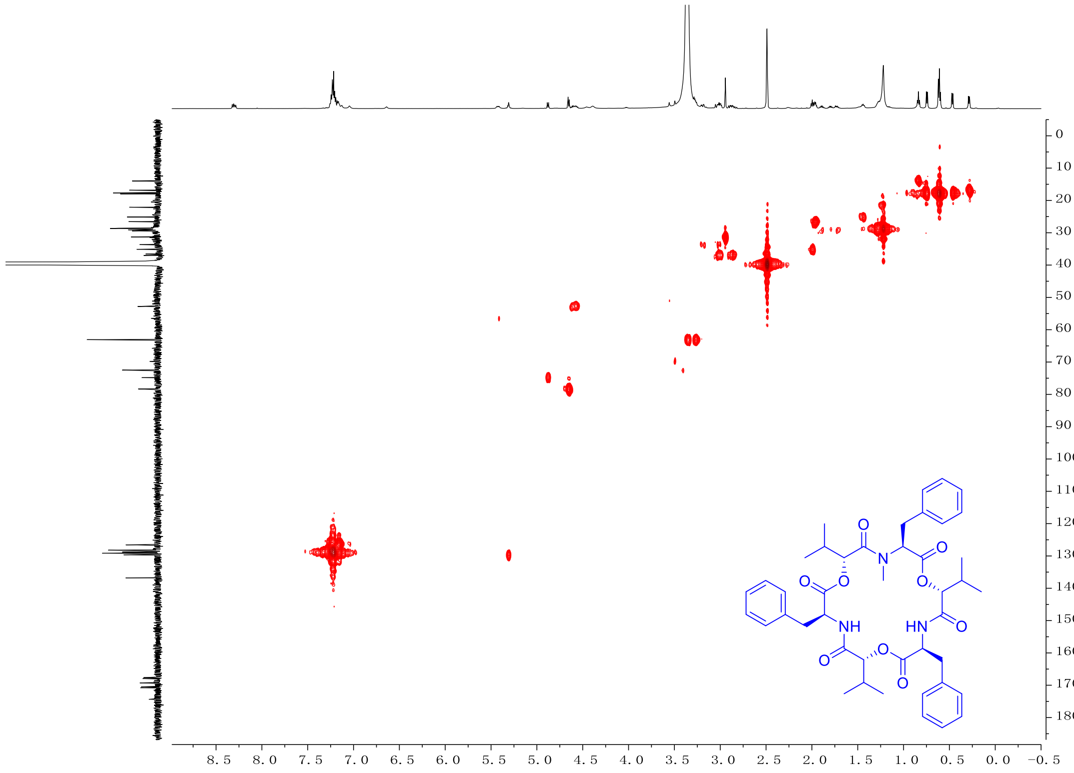

Figure S23. HSQC (600 MHz, DMSO-d_6) spectrum of **2**.

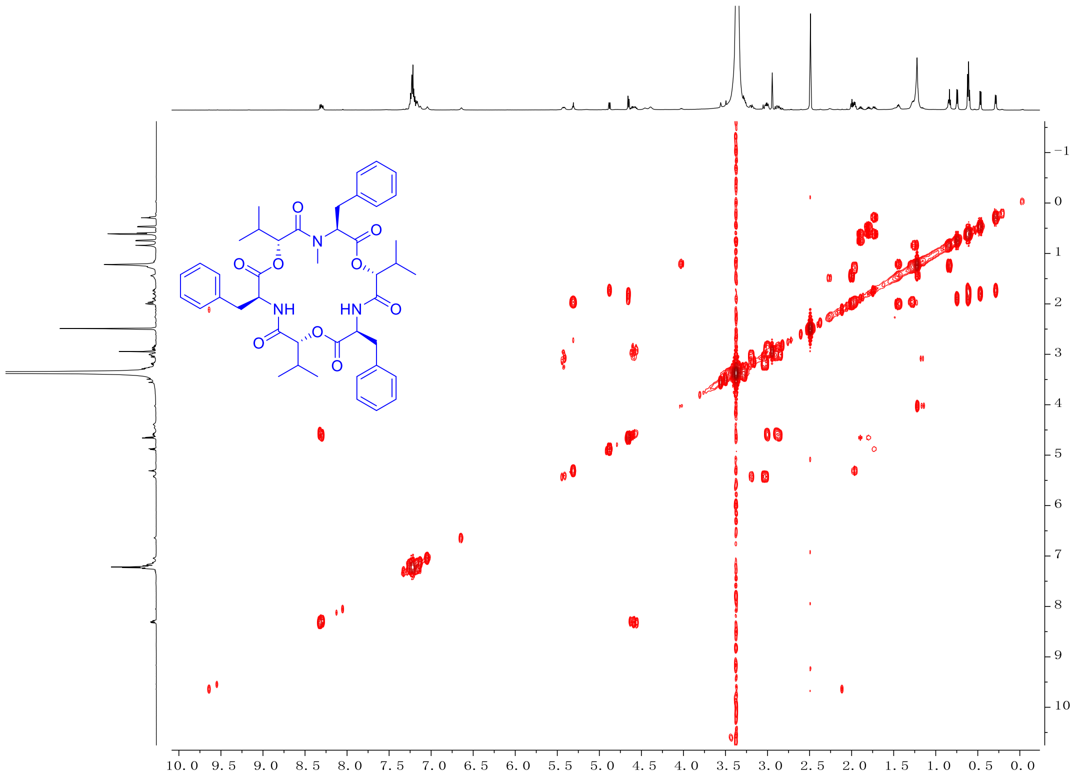

Figure S24. ^{1}H-^{1}H COSY (600 MHz, DMSO-d_6) spectrum of **2**.

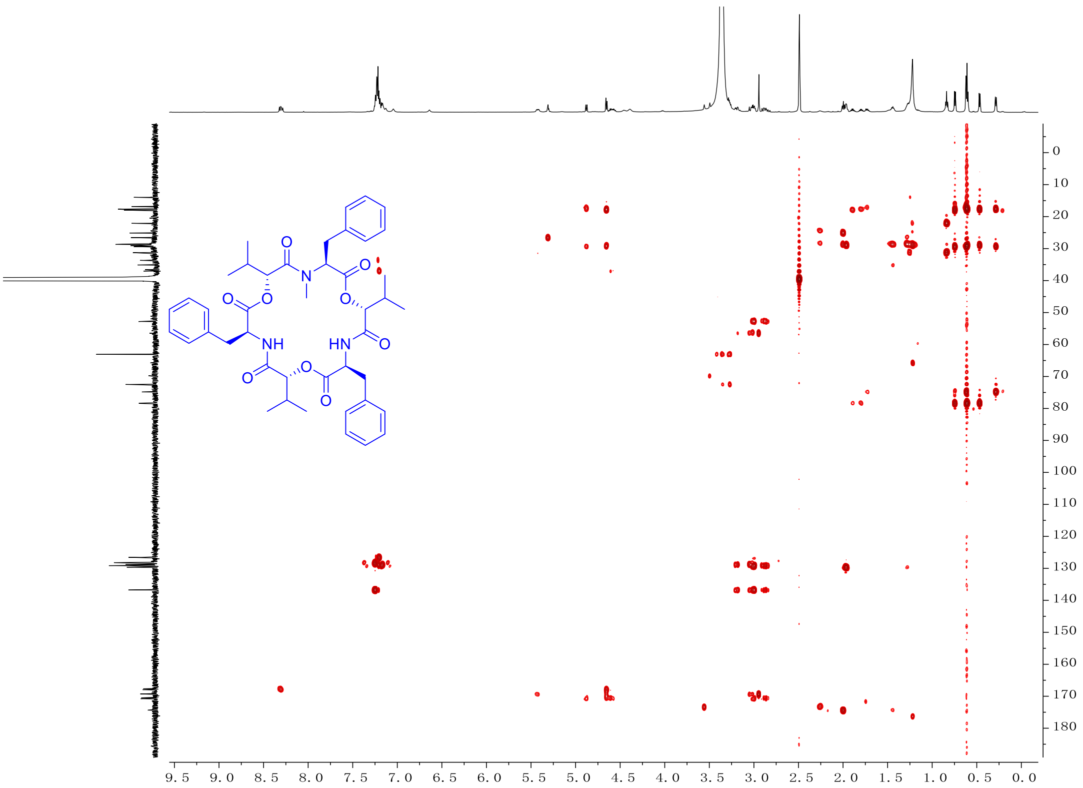

Figure S25. HMBC (600 MHz, DMSO-d_6) spectrum of **2**.

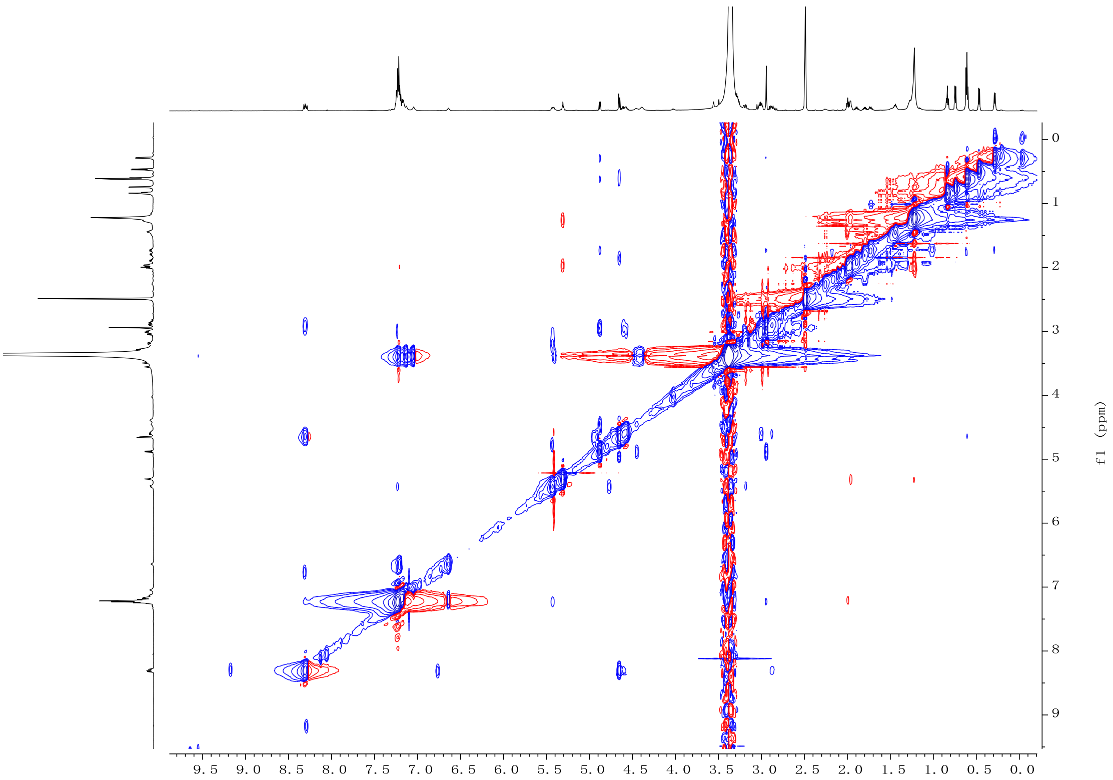

Figure S26. NOESY (600 MHz, DMSO-d_6) spectrum of **2**.

6. Spectral information of 3

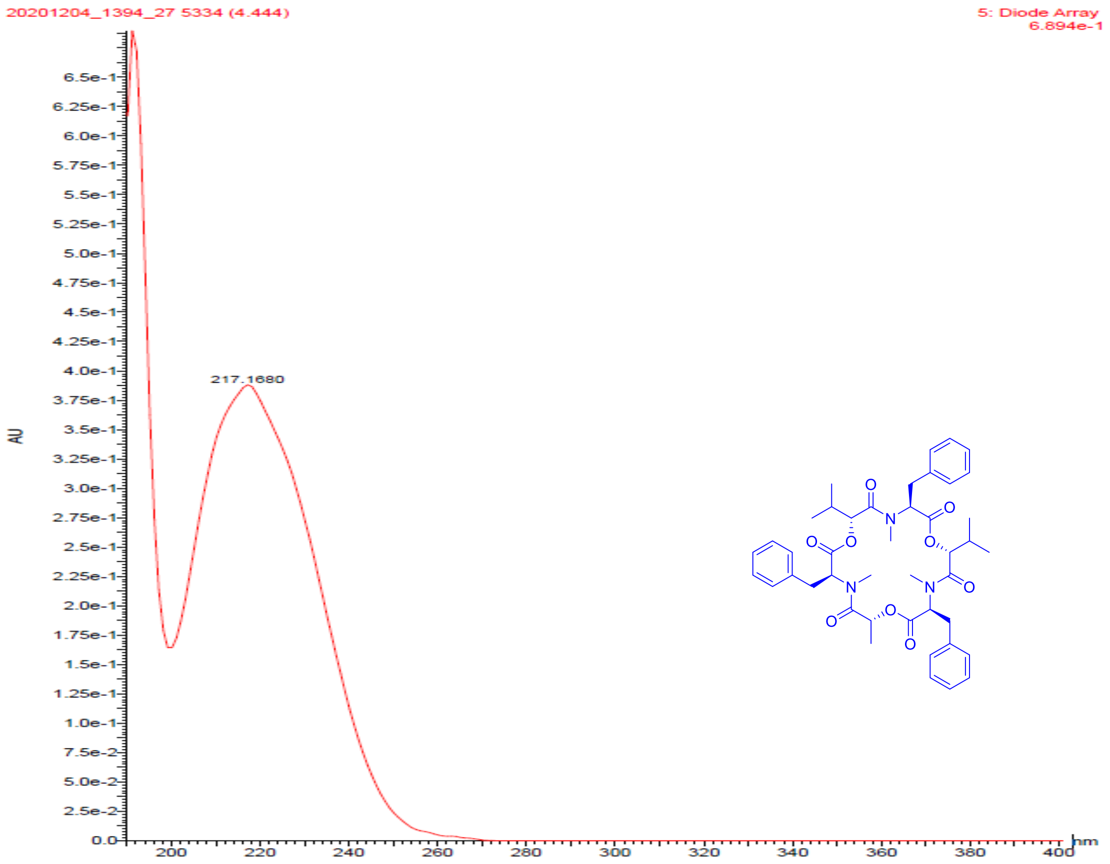

Figure S27. UV spectrum of **3** in MeOH.

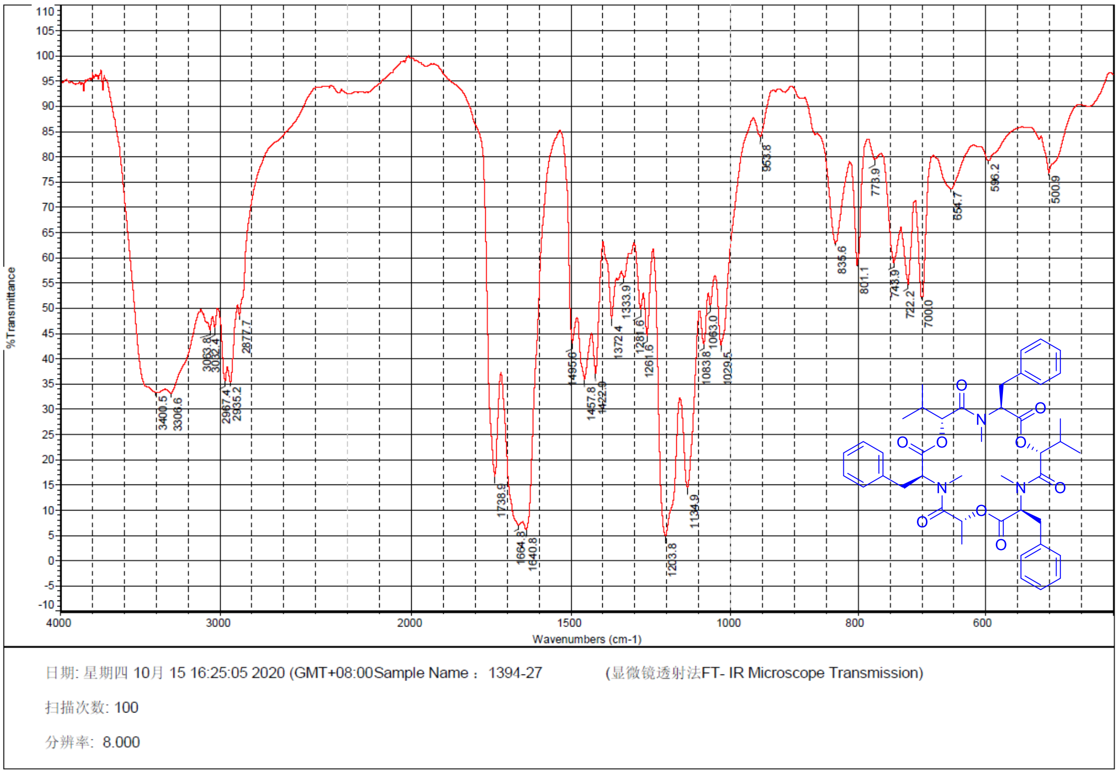

Figure S28. IR spectrum of **3**.

Single Mass Analysis
Tolerance = 5.0 PPM / DBE: min = -1.5, max = 50.0
Element prediction: Off
Monoisotopic Mass, Even Electron Ions
1 formula(e) evaluated with 1 results within limits (all results (up to 1000) for each mass)
Elements Used:
C: 43-43 H: 54-54 N: 3-3 O: 9-9 Na: 0-1

Mass	Calc. Mass	mDa	PPM	DBE	Formula	C	H	N	O	Na
756.3830	756.3860	-3.0	-4.0	18.5	C43 H54 N3 O9	43	54	3	9	

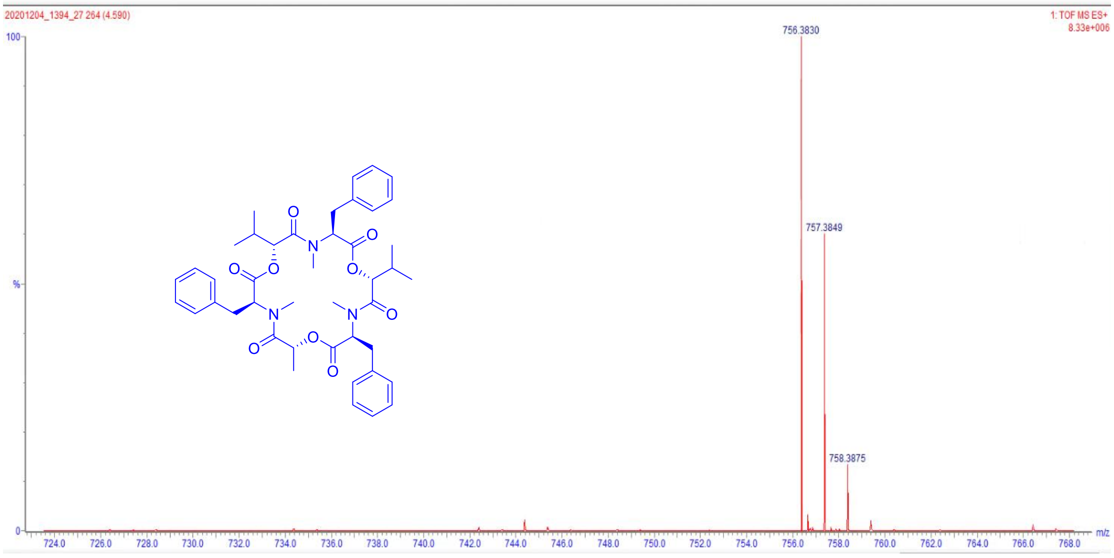

Figure S29. HR-ESIMS spectrum of **3**.

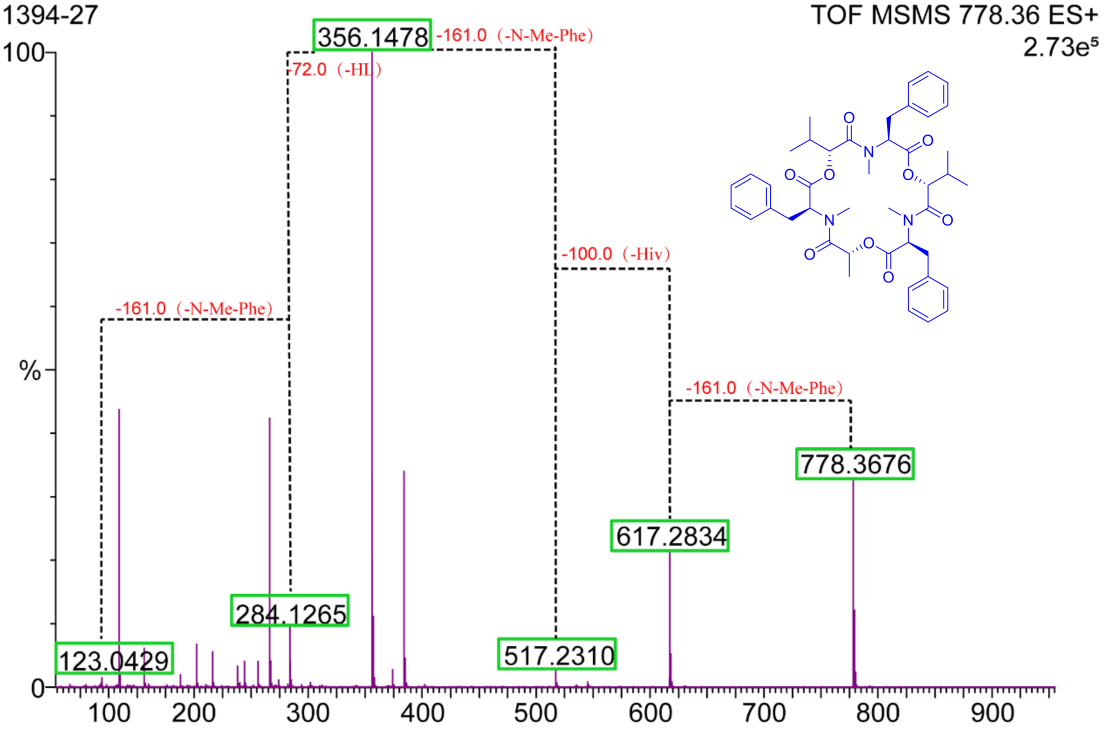

Figure S30. Fragments observed for **3** by HRESIMS/MS.

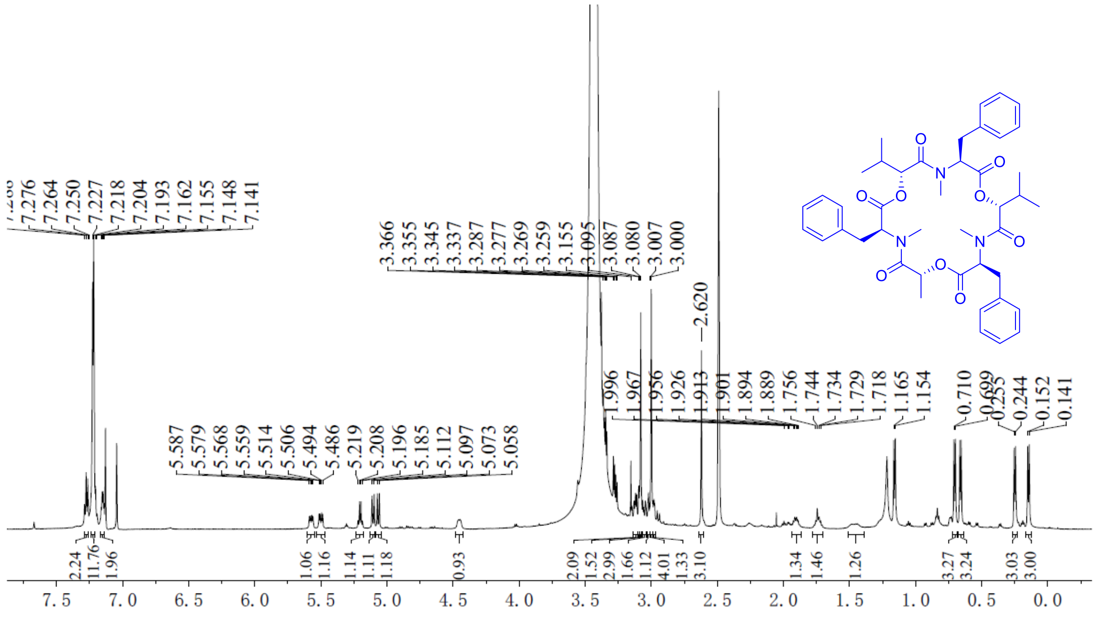

Figure S31. ^{1}H NMR (600 MHz, DMSO-d_6) spectrum of **3**.

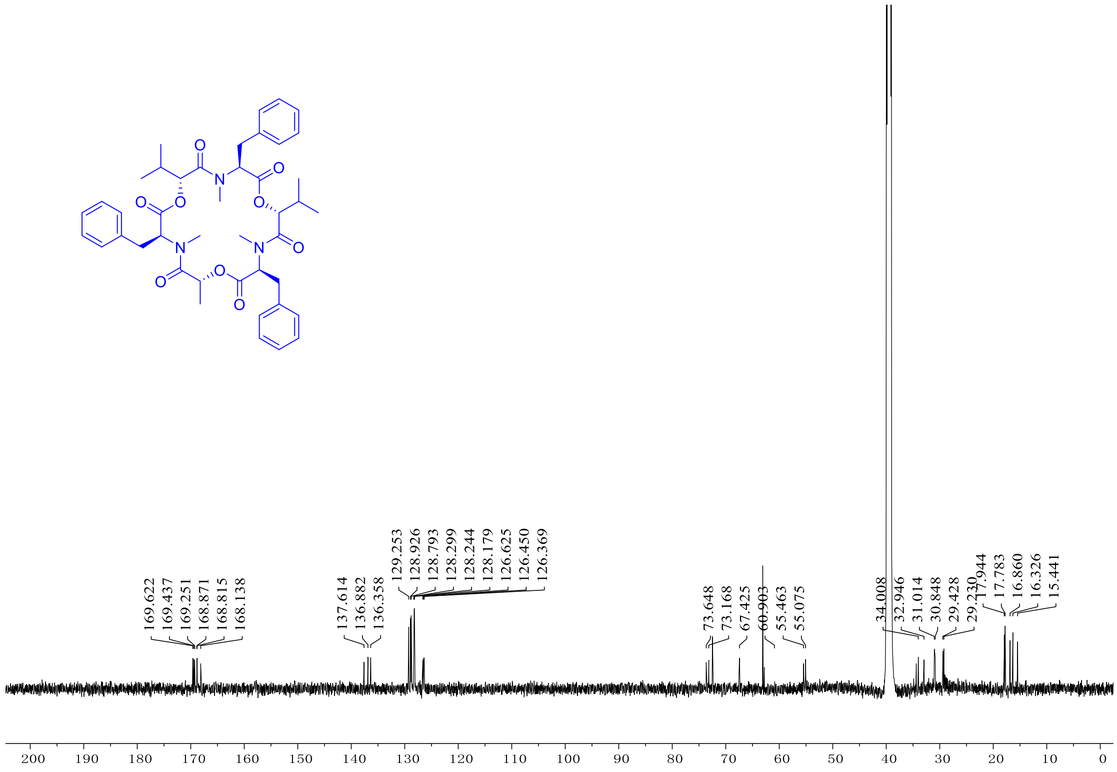

Figure S32. ^{13}C NMR (150 MHz, DMSO-d_6) spectrum of **2**.

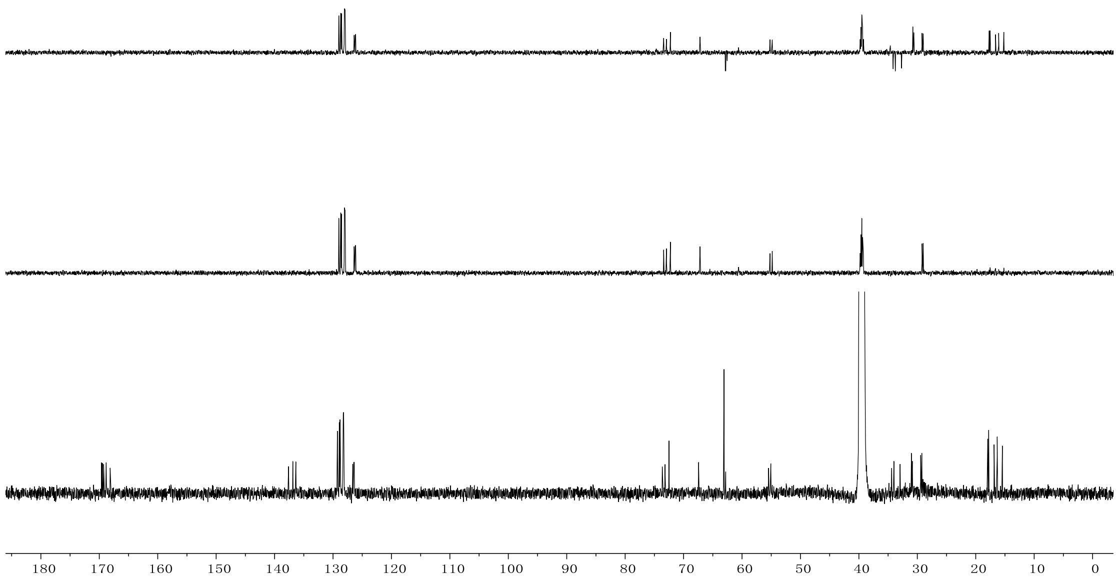

Figure S33. DEPT (150 MHz, DMSO-d_6) spectrum of **3**.

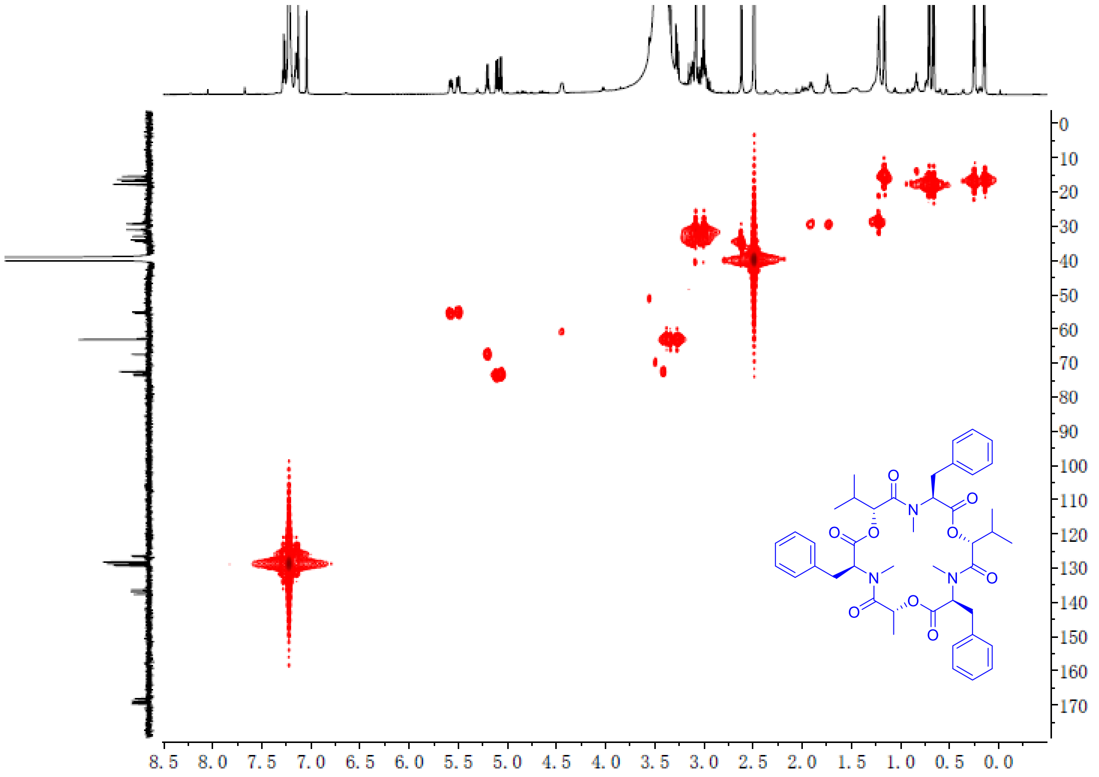

Figure S34. HSQC (600 MHz, DMSO-d_6) spectrum of **3**.

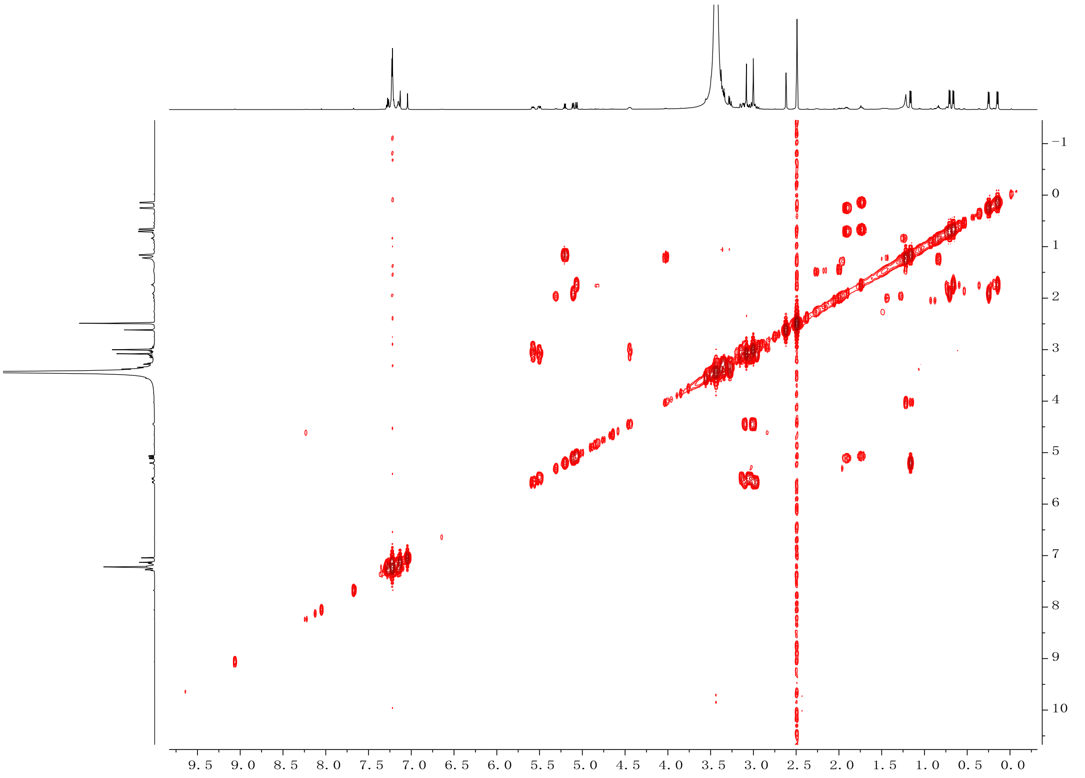

Figure S35. ^{1}H-^{1}H COSY (600 MHz, DMSO-d_6) spectrum of **3**.

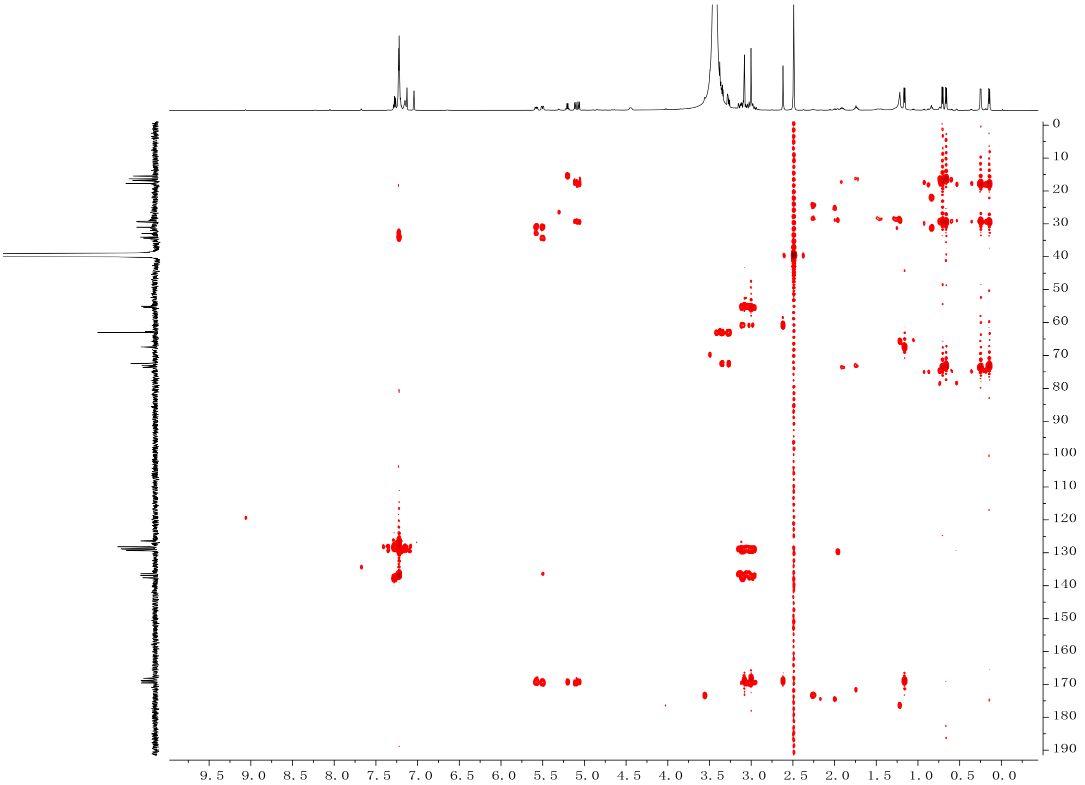

Figure S36. HMBC (600 MHz, DMSO-d_6) spectrum of **3**.

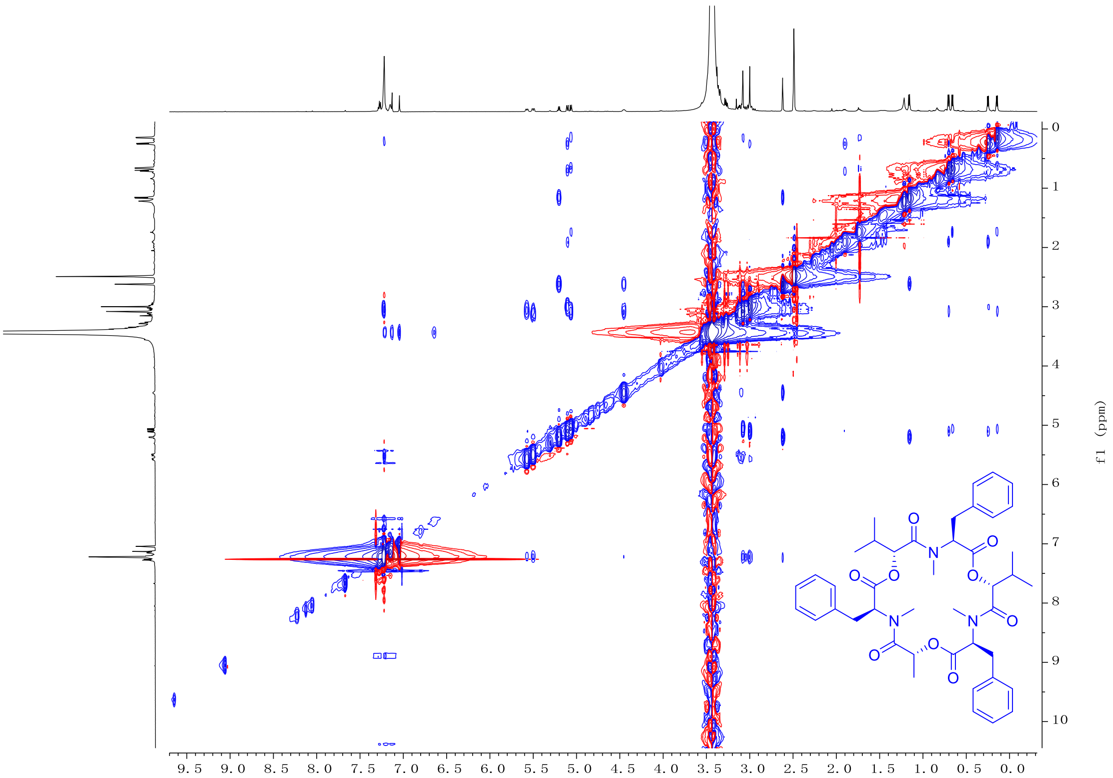

Figure S37. NOESY (600 MHz, DMSO-d_6) spectrum of **3**.
